# Wide Bandgap Semiconductors for Ultraviolet Photodetectors: Approaches, Applications, and Prospects

**DOI:** 10.34133/research.0385

**Published:** 2024-05-27

**Authors:** Fa Cao, Ying Liu, Mei Liu, Zeyao Han, Xiaobao Xu, Quli Fan, Bin Sun

**Affiliations:** ^1^ State Key Laboratory of Organic Electronics and Information Displays, Institute of Advanced Materials (IAM), School of Material Science and Engineering, Nanjing University of Posts and Telecommunication (NJUPT), Nanjing210023, P. R. China.; ^2^ School of Electronic Science and Engineering, Southeast University, Nanjing 210000, P. R. China.

## Abstract

Ultraviolet (UV) light, invisible to the human eye, possesses both benefits and risks. To harness its potential, UV photodetectors (PDs) have been engineered. These devices can convert UV photons into detectable signals, such as electrical impulses or visible light, enabling their application in diverse fields like environmental monitoring, healthcare, and aerospace. Wide bandgap semiconductors, with their high-efficiency UV light absorption and stable opto-electronic properties, stand out as ideal materials for UV PDs. This review comprehensively summarizes recent advancements in both traditional and emerging wide bandgap-based UV PDs, highlighting their roles in UV imaging, communication, and alarming. Moreover, it examines methods employed to enhance UV PD performance, delving into the advantages, challenges, and future research prospects in this area. By doing so, this review aims to spark innovation and guide the future development and application of UV PDs.

## Introduction

**Fig. 1.**
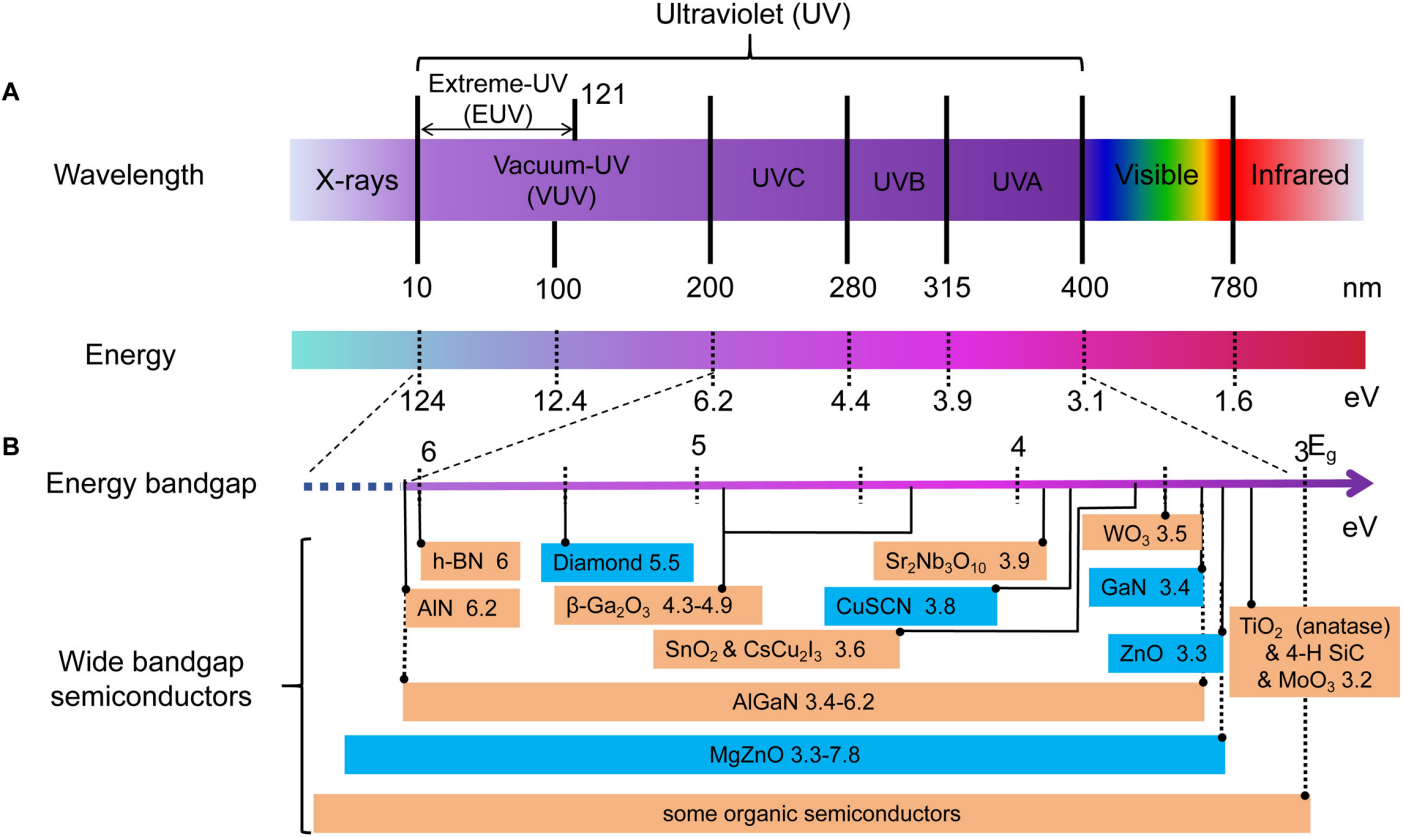
(A) UV spectral region and its subdivisions. (B) Common wide bandgap semiconductors and their bandgaps.

Ultraviolet (UV) light is part of the electromagnetic waves radiated by sunlight, and it is a double-edged sword to human beings. Typically, its wavelengths in vacuum span within the range of 10 to 400 nm, corresponding to energies ranging from 3.1 to 124 eV (Fig. 1A) [1–3]. UV radiation below 200 nm, known as vacuum UV (VUV), can solely propagate in vacuum, while the range between 10 and 121 nm is termed extreme-UV (EUV), widely employed in UV lithography. Only UV light with wavelengths of 200 to 400 nm can penetrate the Earth’s atmosphere. Considering the varied physiological impacts of UV radiation, the 200- to 400-nm range is further divided into UVA (315 to 400 nm), UVB (280 to 315 nm), and UVC (200 to 280 nm) radiation. Controlled exposure to UV light can be beneficial for sterilization, disinfection, bone development, and mineral formation in the body. However, excessive exposure can lead to skin aging and, in severe cases, cancer. Given that UV light lies beyond the human eye’s observation range, the development of various UV sensors aids in the detection and utilization of UV light for human benefit [4–6].

**Fig. 2.**
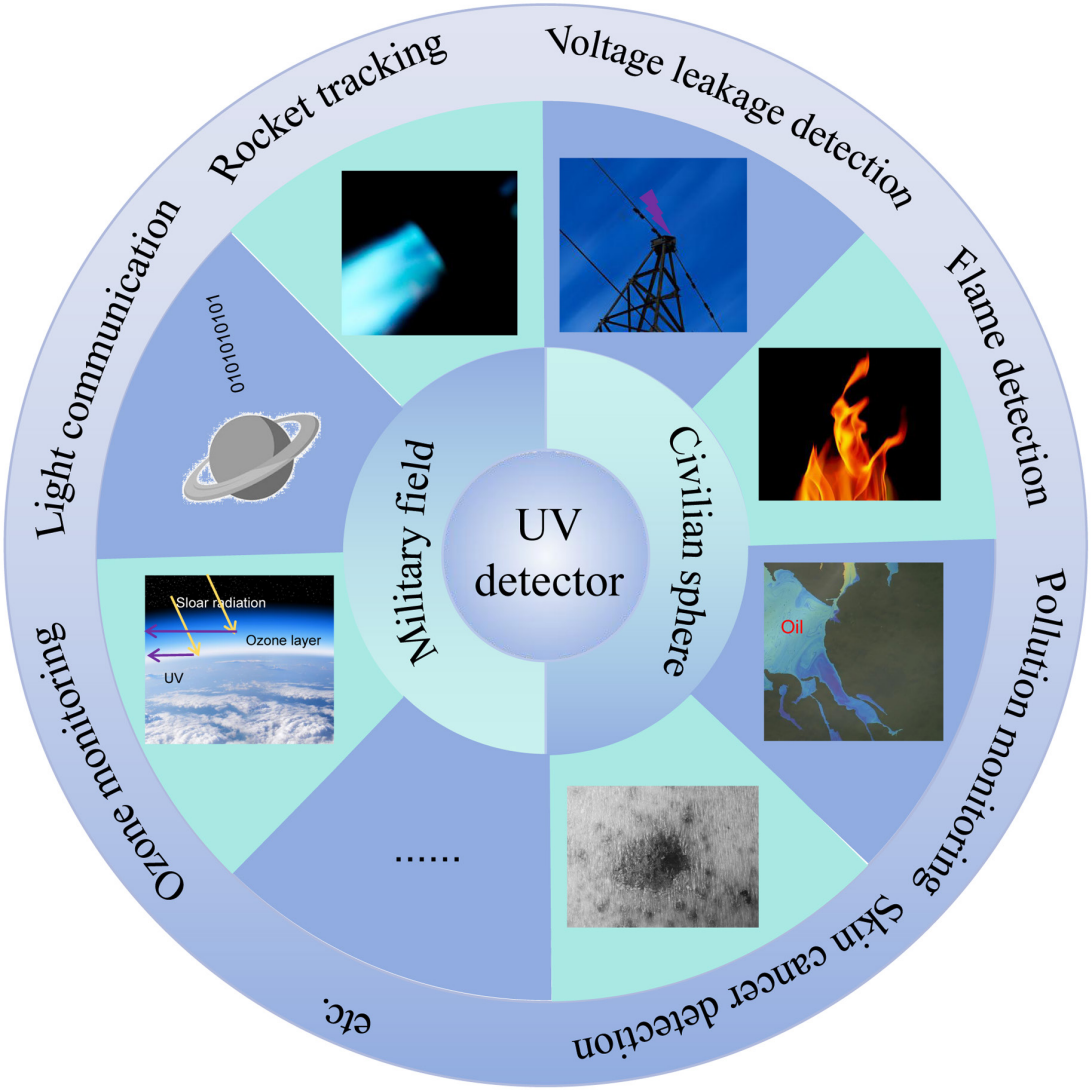
The application of UV PDs in different fields.

The evolution of UV detectors has progressed through three marked stages: vacuum tube photoelectric sensors and photomultiplier tubes, silicon-based UV photodiodes, and the latest wide bandgap semiconductor-based detectors. Photomultiplier tubes, due to their large size, high power consumption, and need for high-voltage operation, are primarily utilized in military applications. On the other hand, silicon-based UV detectors, while widely used, are prone to aging after irradiation and require costly filters to differentiate UV from visible light, increasing overall expenses [7]. Recognizing the limitations of these earlier generations, researchers have turned to the third generation of UV detectors, leveraging advancements in nanotechnology. These wide bandgap semiconductor-based nanomaterials possess large band gaps, such as TiO_2_, 4 H-SiC, ZnO, GaN, SnO_2_, CsCu_2_I_3_, Sr_2_Nb_3_O_10_, β-Ga_2_O_3_, diamond, AlN, and h-BN (Fig. 1B) , ranging from approximately 3.2 to 6 eV. This characteristic allows them to effectively filter out visible light without the need for additional filters [8–22]. Moreover, their photoconductive and photovoltaic properties overcome the drawbacks of previous generations, paving the way for high-performance, low-power, and cost-effective UV detection devices. These advancements hold promise for applications in diverse fields, including military operations such as UV communication, missile detection and tracking, and astronomy, as well as civilian applications like disaster prediction, fire detection, marine pollution monitoring, and biomedical research (Fig. 2) [23–27].

This review aims to fill a notable gap in the literature by offering a comprehensive exploration of methods to enhance the performance of UV photodetectors (PDs) and their wide-ranging applications. While some previous reviews have touched upon wide bandgap semiconductor UV PDs, they often focus on specific materials or aspects, leaving room for a more thorough examination [1–5]. The review commences with a detailed overview of the types and performance parameters of UV PDs, providing a solid foundation for understanding their capabilities. It then delves into the various methods commonly employed to improve their performance, offering valuable insights for researchers and practitioners alike. Additionally, the review examines a wide array of applications for UV PDs, including UV imaging, communication, and alarming, illustrating their versatility and practical utility. Looking ahead, the review offers a forward-looking perspective on the future development prospects of wide bandgap UV PDs, considering advancements in materials, properties, and potential applications. By providing a comprehensive synthesis of current knowledge and future directions, this review aims to serve as a valuable resource for advancing UV PD technology.

## PDs’ Parameters

### Responsivity (*R*)

Responsivity describes the ability of converting photons to electrons of a PD. When irradiated by incident light, photocurrent (*I_l_*) and dark current (*I_d_*) are both generated. Thus, *R* can be defined as follows [28–30]:

$$R=\frac{I_{l}-I_{d}}{\mathrm{PA}}$$ (1)

which is related to the power density (*P*) of incident light due to the bandgap and surface recombination of the active layer (*A*).

### External quantum efficiency

External quantum efficiency (EQE) is the ratio of the number of collected photogenerated carriers to the number of incident photons, showing the conversion capability of photon to carrier, which can be described as follows [31–33]:

$$\text{EQE}=\frac{\mathrm{Rhc}}{e\lambda }$$ (2)

where *h* is the Planck constant, *c* represents the speed of light, *e* is the electron charge, and *λ* means the incident wavelength.

### Noise equivalent power

Noise equivalent power (NEP) is the signal power irradiated on the PD when S/N (signal to noise ratio, which means the ratio of signal peak value to the noise effective value) = 1, representing the ability of detecting weak signals [6,34–36]. Considering that the noise level is proportional to the square root of the measured bandwidth, NEP is defined at the bandwidth = 1 Hz, which can be described as below:

$$\text{NEP}=\frac{I_{\text{noise}}}{R}$$ (3)

where *I*_noise_ is the noise current at bandwidth of 1 Hz.

### Specific detectivity (D*)

Detectivity is a NEP-related parameter, describing the detecting sensitivity of PD from a noise background [37–40]. When normalized by the PD area, the detectivity is defined as D^*^, which is shown below:

$$D^{*}=\frac{{\left(S∆f\right)}^{0.5}}{\text{NEP}}$$ (4)

Normally, the noise current consists of shot noise, thermal noise, flicker noise (1/*f* noise), and generation-recombination noise. When the shot noise dominates the contribution, D^*^can be simplified as follows:

$$D^{*}=\frac{R}{{\left(2eI_{d}/S\right)}^{0.5}}$$ (5)

in which *S* is the photosensitive area.

### Response time (*t*)

Response time manifests the reaction of the PD to a certain signal, in order to keep pace with the incident light signal, the PD should possess a quick response speed. The response time τ contains the rise time (*t*_rise_) and decay time (*t*_decay_). Generally, *t*_rise_ and *t*_decay_ are defined as the time of the response current rising (or falling) from 10% to 90% (or 90% to 10%) of the maximum response current [41–43].

Besides, from the view of frequency domain, *R* of PD is also decided by the time constant *t*, which can be described as follows:

$$R\left(f\right)=\frac{R_{0}}{\sqrt{1+{\left(2\mathrm{pft}\right)}^{2}}}$$ (6)

where R_0_ stands for the *R* of the PD irradiated by constant light signal and *f* is the frequency of the incident light. When the *R* intensity decreases to 0.707*R_0_*, the corresponding *f* is cutoff frequency *f*_*c*._ Thus, it is clear that a shorter response time could lead to a higher cutoff frequency.

### On/off ratio

The on/off ratio, often referred to as the light-to-dark current ratio, is commonly defined as the ratio of the light current of the PD to the dark current (*I*_light_/*I*_dark_). Alternatively, it could be expressed as the ratio of photogenerated current to the dark current (∆*I*/*I*_dark_). This ratio serves as an indicator of a detector’s sensitivity to signals of detectors [44–46]. It is important to note that the on/off ratio is easily affected by the intensity and wavelength of incident light; therefore, precise control of measurement conditions is essential. Moreover, achieving a higher on/off ratio, which leads to enhanced contrast in final imaging, requires optimization of photoelectric conversion and charge transportation, as well as suppression of defects.

### Linear dynamic range

Linear dynamic range (LDR) is a vital figure of merit of detector, implying the range of signal intensity in which the PD could be precisely distinguished. In the LDR range, the photogenerated current demonstrates a linear dependence on the light intensity, which can be defined as follows [47–49]:

$$\text{LDR}=20\text{log}\frac{P_{\text{max}}}{P_{\text{min}}}$$ (7)

where *P*_max_ and *P*_min_ represent the largest and lowest light intensity of the linear range, respectively. In the sensing or imaging industry, a high LDR means a broad latitude, laying the foundation for high-fidelity imaging. In some cases, the LDR can be calculated as:

$$\text{LDR}=20\text{log}\frac{I_{\text{max}}}{I_{\text{min}}}$$ (8)

where *I*_max_ and *I*_min_ represent the photocurrent and dark current, respectively, under a light intensity of 1 mW/cm^2^.

### Spectral selectivity

For most PDs, they typically respond to a specific wavelength range, such as UV light, visible light, or infrared light. The spectral selectivity is often evaluated by the full width at half maximum (FWHM) of the detector’s response. A narrower FWHM allows the detector to differentiate complex spectrum signals, enhancing spectral selectivity. In many cases, PDs with excellent spectral selectivity are achieved through the absorption properties of their active layers or device configuration design [50].

### Cutoff wavelength

The cutoff wavelength of the PD refers to the longest wavelength (λ_0_) that can be detected by the PD, which is related to the energy band (E_g_) structure of the material.

$$\lambda_{0}=\frac{\mathrm{hc}}{E_{g}}\;$$ (9)

## The Classification of UV PDs

**Fig. 3.**
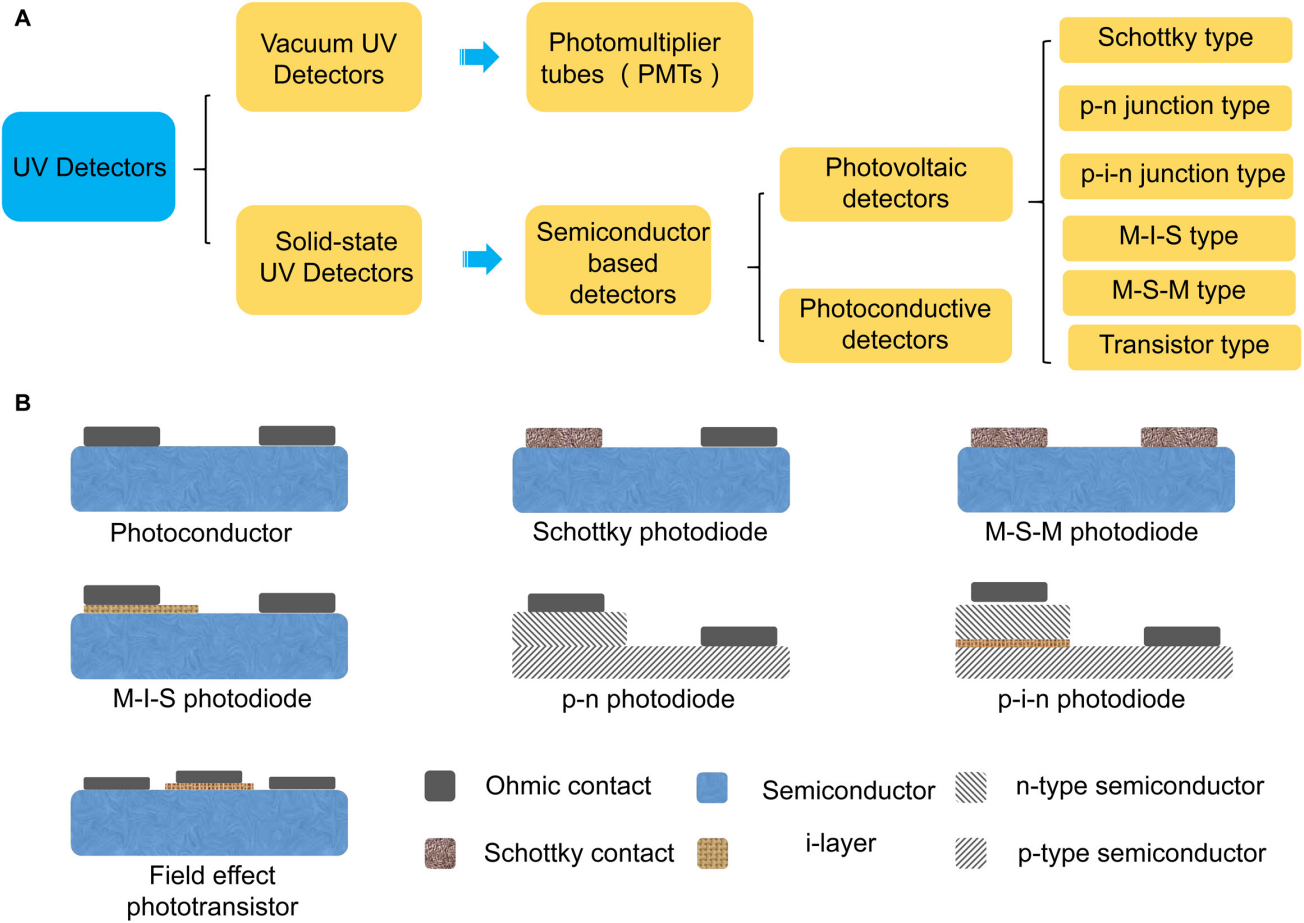
(A) Classification of UV PDs. (B) Schematic structure of the different types of UV PDs.

In general, UV PDs can be categorized into two types: vacuum and solid-state devices (Fig. 3A). VUV PDs, relying on photomultiplier tubes, have a long-established presence. However, their drawbacks include heavy weight, high power consumption, and the need for high operating voltages, limiting their widespread use. To address these limitations, solid-state UV PDs have been developed, primarily using wide bandgap semiconductor materials. Moreover, wide bandgap semiconductors can be further classified into photoconductive PDs (photoconductors) and photovoltaic PDs. Examples of the latter include Schottky photodiode, MSM (metal-semiconductor-metal, both of the two electrodes are Schottky contact with the semiconductor) photodiode, MIS (metal-insulator-semiconductor) photodiode, pn photodiodes, pin photodiodes, and field effect phototransistors (Fig. 3B). The advantages and drawbacks of these various PDs have been extensively discussed in previous reports [51–54]. This classification scheme provides a comprehensive understanding of the strengths and weaknesses inherent in each type of UV PD.

## Materials for UV PDs

UV PDs based on wide bandgap semiconductors offer several advantages, including small size, light weight, affordable price, and excellent UV response ability, making them an attractive option for UV detection [55–60]. Additionally, wide bandgap semiconductors allow UV PD to operate at room temperature without visible light interference. Over the past two decades, significant progress has been made in the development of various wide bandgap semiconductor materials for UV PDs. Commercially available options include GaN, SiC, and diamond-based UV PDs. To meet diverse demands, researchers are exploring new wide bandgap semiconductor materials. For example, various metal oxide (such as ZnO, TiO_2_, SnO_2_, WO_3_, MoO_3_, ZnS, and Ga_2_O_3_) UV PDs have been developed due to their ease of fabrication and low cost. Additionally, some two-dimensional (2D) and organic wide bandgap semiconductors (such as h-BN, Sr_2_Nb_3_O_10_, and Ca_2_Nb_3_O_10_) are being investigated for flexible and wearable UV PDs [15,61–65]. Furthermore, newly emerging wide bandgap perovskite (CsCu_2_I_3_, Cs_3_Cu_2_I_5_, CsAg_2_I_3,_ etc. [28].)-based UV PDs are gaining attention due to their multifunctional properties. In Table, we gave the main parameters of the recently reported UV PDs based on both traditional and newly emerging wide bandgap semiconductors.

**Table.** Figure of merits of the main parameters of various wide bandgap semiconductor-based UV PDs.

	Materials	λ (nm)	Bias (V)	R (mA/W)	D^*^(Jones)	Rise/fall time	On/offratio	Reference
ZnO(MgZnO)	ZnO/Au/Al_2_O_3_	365	0	6.8	1.7 × 10^9^	0.16/0.16 s	805	[66]
	Plasmonic Ag/ZnO	380	5	2,860	-	15/330 s	-	[67]
	ZnO/CdMoO_4_/Au	350	5	377	-	16/9.2 s	377	[68]
	Plasmonic Cu/ZnO	365	10	2.3 × 10^5^	-	7.4/29	~3	[69]
	ZnO:Na/ZnO:Al	360	0	-	-	82/82 ms	143	[70]
	BeZnO	240	0	2 × 10^−3^	-	35/880 us	<100	[71]
	MgZnO/PANI	260	0	0.16	-	-	-	[25]
	MgZnO/ZnO	350	4	~10^3^	-	-	-	[72]
GaN(AlGaN)	AlGaN/GaN	265	15	2.7 × 10^7^	5 × 10^8^	30/55.4 ms	-	[73]
	GaN/Ga_2_O_3_	254	0	28.4	-	0.1/0.08 s	-	[74]
	GaN	365	3	51.6	-	0.08/0.16 s	-	[75]
	GaN/MXene	355	0	284	7 × 10^13^	7.5/1,670 μs	-	[76]
	GaN	325	−2	2.8 × 10^4^	7.8 × 10^10^	2.8/3.6 ms	-	[77]
	n-GaN/p-β-Ga_2_O_3_	245	0	1.45	1.6 × 10^11^	3.5/30 μs	-	[26]
	GaN/ZnO/Graphene	360	0	6	-	-	-	[78]
TiO_2_	TiO_2_/NiO/TiO*_x_*	380	0	6	-	-	-	[79]
	TiO_2_/BTO	310	1	8.5	6 × 10^10^	-	10^2^	[80]
	TiO_2_/BiOCl	350	−5	4.1 × 10^4^	1.4 × 10^14^	12.9/0.8 s	2 × 10^5^	[81]
	TiO_2_/Si	365	−4	6.7 × 10^3^	1.3 × 10^12^	~0.1/0.1 ms	39.6	[56]
	TiO_2_/In_2_O_3_	330	−5	3.2 × 10^5^	2.9 × 10^16^	0.26/0.13 s	-	[82]
	Au/TiO_2_/P_3_HT	350	0	0.25	2.9 × 10^10^	0.5/2.1 s	-	[83]
Ga_2_O_3_	Ga_2_O_3_	260	10	6.6 × 10^3^	>10^14^	-	>10^4^	[84]
	β-Ga_2_O_3_/PANI	246	0	21	1.5 × 10^11^	0.3/8.1	-	[85]
	n-Ga_2_O_3_/p-CuSCN	254	5	13.3	-	62/35 ms	-	[86]
	Graphene/Ga_2_O_3_	254	10	2.9 × 10^5^	1.7 × 10^15^	1.7/26.8 s	-	[87]
	β-Ga_2_O_3_	254	0	10.8	-	0.6/0.4 s	<10	[88]
	α-Ga_2_O_3_/PEDOT:PSS	245	0	1.4	-	0.5 s	-	[89]
	β-Ga_2_O_3_	254	1	-	-	0.6/8.9 s	-	[90]
SnO_2_	SnO_2_/CuZnS	315	0	1.5	-	45/1,100 μs	180	[91]
	SnO_2_/CuI	300	0	8.9	1.9 × 10^12^	0.3/1.1 ms	>10^5^	[92]
	SnO_2_	322	10	1.3 × 10^6^	5.4 × 10^14^	80/80 ms	1,630	[93]
	SnO_2_/Perovskite	-	0.42	-	1.1 × 10^13^	75/40 ms	-	[94]
	n-SnO_2_/p-GaN	360	0	185	10^13^	0.3/61 μs	-	[95]
	SnO_2_	300	−5	10^7^	-	33/85 s	-	[96]
SiC	SiC	375	0.6	218.7	2.2 × 10^13^	17/48 ms	-	[97]
	4-H SiC	375	5	824	6 × 10^10^	0.52/0.88 s	-	[98]
	SiC	445	0.5 mV	12.2	1.1 × 10^9^	0.39/0.56 s	-	[99]
	B doped 3C SiC	405	5	10^5^	6.8 × 10^14^	0.05/0.05 s	-	[100]
	SiC/MoS_2_	365	20	5,000	~2 × 10^10^	0.58/0.33 s	-	[101]
Perovskite	CsAg_2_I_3_	265	2	0.355	-	23/43 ms	-	[77]
	CsCu_2_I_3_	360	3	276.9	1.3 × 10^12^	0.37/1.1 ms	1,570	[102]
	Cs_3_Bi_2_Br_9_/Cs_3_BiBr_6_	360	0	59.4	1.2 × 10^12^	0.2/1.09 μs	18,881	[103]
	CsPbCl_3_	375	0.5	3.3 × 10^4^	4.2 × 10^12^	0.2/0.4 ms	5 × 10^5^	[104]
	Cs_3_Cu_2_I_5_	270	5	3,780	-	0.16/0.20 s	-	[105]
	Sr_2_Nb_3_O_10_	270	1	1.2 × 10^6^	1.4 × 10^14^	0.4/40 ms	-	[106]
	Ca_2_Nb_3_O_10_	300	5	80	1.1 × 10^12^	0.12/1.2 ms	4 × 10^3^	[107]
	CaNb_2_O_6_	375	1	-	-	0.8/0.9 ms	~2	[108]
	SrTiO_3_	400	−2	-	-	7/115 μs	-	[109]
Others	Sm_2_O_3_/Graphene/SiC	255	0	19.8	1.2 × 10^11^	20/40 ms	-	[110]
	Si/Yb_2_O_3_/Graphene	255	0	9.8	2.5 × 10^10^	87/73 ms	-	[111]
	MoO_3-x_	380	3	1,537	-	0.1/0.1 s	-	[112]
	WO_3_	320	1	329	-	<40/<40 μs	-	[113]
	BiOCl/ZnO	350	5	182.2	-	29/11 s	798	[114]
	AlN/ZnO	193	1	3.8 × 10^5^	-	0.12/0.36 s	-	[115]
	h-BN	220	20	3.4	3.9 × 10^10^	0.12/0.16 s	-	[116]
	Diamond	225	5	5.9 × 10^5^	3.4 × 10^15^	0.16/120 μs	-	[117]
	TCTA:C_60_	280	−5	-	6 × 10^12^	2.7/3.3 ms	-	[118]
	TAPC:C_60_	335	−14	1.2 × 10^6^	1.3 × 10^14^	0.4/1.0 s		[119]

## The Approaches for Improving UV PDs’ Performance

With the increasing demands for the performance of UV PDs in modern military and civil applications, meeting the “5 S” requirements is crucial: high sensitivity, high signal-to-noise ratio, spectral selectivity, fast response speed, and high stability [33,105,120–122]. UV PDs based on a single wide bandgap semiconductor material may not fulfill all these diverse purposes. For example, specific scenarios may call for large photocurrent, small dark current, fast response speed, high sensitivity, or without energy consumption. To address these varied requirements, researchers have devised numerous approaches, including surface plasmon resonance, nanostructure design, surface/interface carrier transport modulation, pyro/ferro/piezo-phototronic effect, and the selection of electrodes and work function adjustment, among others. These methods aim to enhance UV PD performance through tailored strategies. Detailed discussions and examples of methods for improving UV PD performance will be presented in the following sections.

### Surface plasmon resonance

**Fig. 4.**
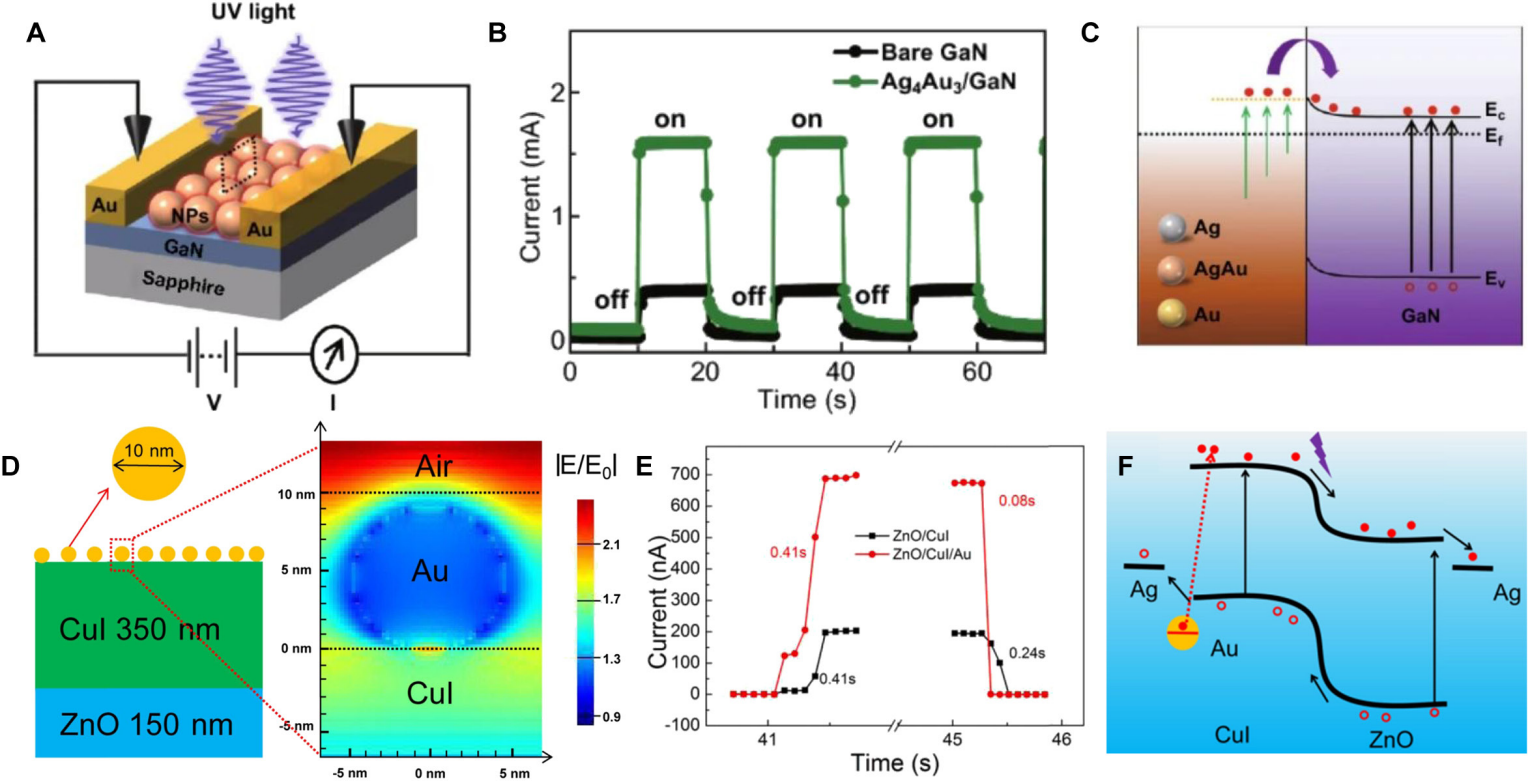
(A) Schematic of plasmonic metallic NPs on GaN. (B) Photoresponse of the GaN with and without plasmonic metallic NPs decorated (@ 0.1 V, 385-nm light). (C) Schematics of the charge transfer process of the plasmonic NP-decorated GaN under UV illumination [127]. Copyright 2020 Springer Nature. (D) FDTD (finite difference time domain) simulation results of Au on CuI layer with excitation of UV light. (E) Current–time (*I*–*T*) curves of ZnO/CuI/Au and ZnO/CuI PD (0 V). (F) Schematics of the charge transfer process of ZnO/CuI/Au PD under UV light [128]. Copyright 2021 Elsevier Ltd.

Plasmons, characterized by the resonances of mobile electrons in metals and other conductive materials, exhibit strong optical responses due to the coherent oscillation of free electrons in space and time [66,123–126]. When metallic nanoparticles (NPs) are coupled with semiconductors, the oscillating free electrons can inject into the semiconductors, offering great potentials for PDs. Numerous studies have been reported, for example, various sizes and compositions of Ag, Au NPs, and AgAu alloy NPs have been synthesized on GaN substrate by the solid-state dewetting methods [127]. Figure 4A illustrates a GaN photoconductor decorated with metallic NPs. Incorporating metallic NPs significantly enhances the photocurrent, reaching up to 112 A/W under specific conditions (0.1-V bias, 385-nm light, 0.03 mW/mm^2^) (Fig. 4B). When exposed to UV light, hot electrons from the metallic NPs transfer to the GaN (Fig. 4C), effectively boosting the device’s photocurrent.

Energy-efficient UV PDs capable of operating without external bias are highly desirable. Utilizing the internal electric field of a pn junction enables the separation of photogenerated electrons and holes, facilitating self-powered photoelectric detection and reducing electromagnetic radiation exposure during idle periods. However, limitations in photocarrier mobility can lead to low responsivity of UV PDs at 0-V bias. In previous work [128], a synergistic effect between plasmonic gold (Au) and the built-in electric field was leveraged to enhance UV photodetection. Under UV light (365 nm) illumination, hot electrons generated in Au NPs transfer to CuI conduction band (CB) (Fig. 4D and F). Subsequently, electrons swiftly move to the corresponding electrode under the built-in electric field, significantly increasing the photocurrent from ~200 nA to ~700 nA and achieving a high self-powered UV responsivity of 61.5 (Fig. 4E). The decoration of plasmonic metal NPs presents an effective strategy for enhancing the photodetect abilities of UV PDs. In UV light condition, these NPs supply abundant hot electrons, thereby augmenting the photocurrent of the PD. Conversely, in dark conditions, photogenerated carriers can transfer to the metal NPs, reducing the decay time of the PD. This dual functionality contributes to improved performance across varying illumination levels, making plasmonic metal NPs a valuable asset in enhancing the overall efficiency and responsiveness of UV PDs.

### Nanostructure design and surface/interface modulation

**Fig. 5.**
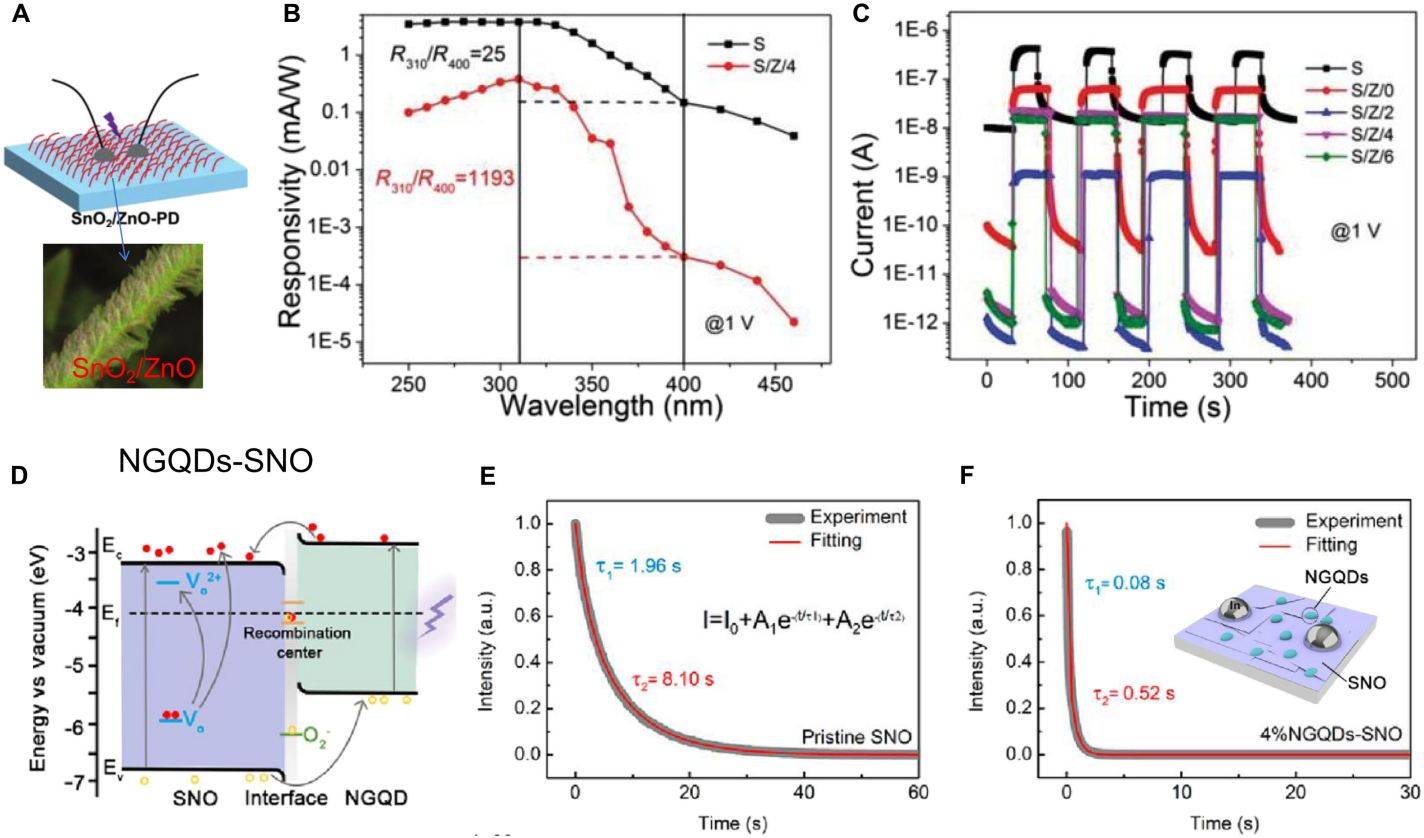
(A) Schematic of SnO_2_/ZnO PD and scanning electron microscopy (SEM) images of a single structure. (B) Spectra response of the PD (@ 1 V). (C) *I*–*T* curves of the PDs [133]. Copyright 2022 WILEY-VCH. (D) Schematic energy band diagrams of the NGQD-SNO PD. (E and F) Decay process of the SNO and the NGQD-SNO PD, and the inset of (F) is the schematic diagram of the NGQD-SNO PD [137] .Copyright 2022 American Chemical Society.

The advancement in nanomaterial technology has revealed the diverse performance capabilities of materials with varying nanostructures [129-132]. For instance, pure wide bandgap semiconductor PDs often struggle with significant dark current issues, resulting in a relatively small UV/visible ratio. To address this, the Fang group [133] devised a pine-branch-like SnO_2_/ZnO heterostructure (Fig. 5A). By incorporating ZnO branches, they managed to reduce the PD’s dark current from 10^−8^ A (SnO_2_) to 10^−12^ A (SnO_2_/ZnO) at 1-V bias (Fig. 5C). Meanwhile, the on/off ratio surged from 42 to 16,405, indicating an enhanced UV/visible ratio (*R*_310_/*R*_400_) of 1,193 (Fig. 5B). With the decoration of ZnO branches, there is a significant increase in device resistance, leading to the reduced dark current as well as the enhanced on/off ratio. The enhanced on/off ratio of SnO_2_/ZnO heterostructure at 310 nm eventually contributes to the UV/visible ratio since the on/off ratio at 400 nm remains almost unchanged. The reduced dark current enables the PD with weak light detecting ability, and the enhanced UV/visible ratio provides the device a better performance in visible-blind UV detection. As we know, when the size of metal oxide nanomaterials is reduced to 2D or 1D level, the surface oxygen defects will increase, which will affect the separation and transmission of photogenerated carriers. For a better UV PDs, the materials with less surface/interface defect are required [134–136]. Liu et al. [137] integrated 2D perovskite Sr_2_Nb_3_O_10_ (SNO) nanosheets with nitrogen-doped graphene quantum dots (NGQDs), forming a unique nanoscale heterojunction (Fig. 5D). With the modulating surface/interface of SNO (the absorption O^2−^ is reduced and the desorption of O^2−^ is facilitated under UV light), the response speed of the carriers is significantly enhanced (Fig. 5E and F). The enhanced response speed endows the UV PD with better imaging ability. Besides, plasma treatment and plasma metal NPs also have the functions of surface/interface modification effect [124].

### Ferro/pyro/piezo-phototronic effect

**Fig. 6.**
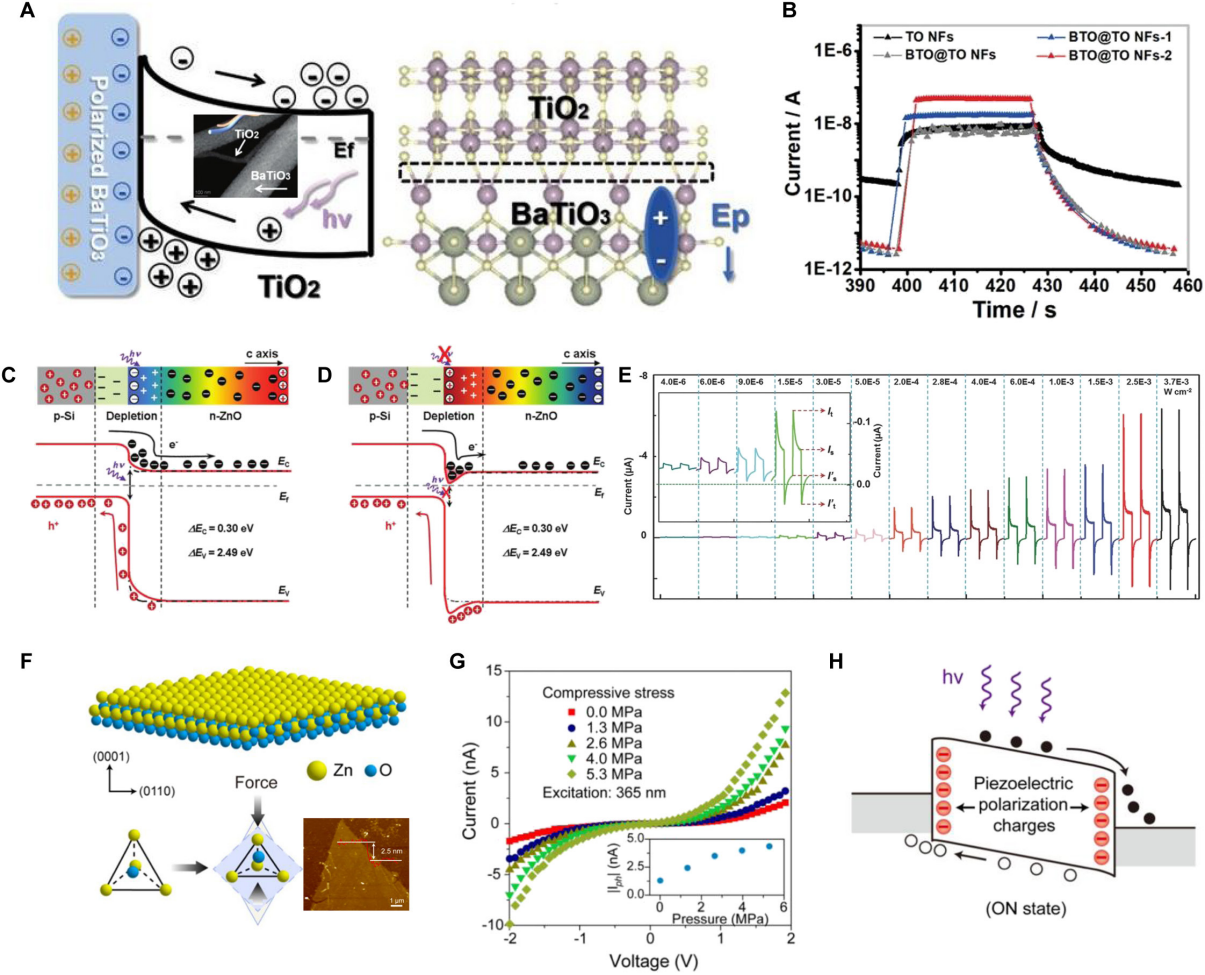
(A) Energy level of the BaTiO_3_/TiO_2_ interface, where the self-polarization in BaTiO_3_ induced an upward band bending of TiO_2_ and the inset is the corresponding SEM images. (B) *I*–*T* curves of TiO_2_ and BaTiO_3_/TiO_2_ PDs [139]. Copyright 2023 WILEY-VCH. (C and D) P-Si/n-ZnO heterojunction with and without UV light to illustrate the working mechanism of pyro-phototronic effect-based PDs. (E) Pyro-phototronic effect enhanced performances of p-Si/n-ZnO UV PD [140]. Copyright 2016 WILEY-VCH. (F) Schematic and atomic force microscopy (AFM) images of ZnO nanosheet with wurtzite structure and its piezoelectricity. (G) *I*–*V* curves of the ZnO devices under different pressure (@ 2 V; 365 nm); 5- and 0-MPa pressure shown in the inset. (H) Band diagrams of the atomically thin ZnO PD under illumination with pressure [142]. Copyright 2021 Elsevier Ltd.

Ferroelectric materials exhibit spontaneous polarization, which can be reversed under an external electric field. This property holds immense promise for tuning interfacial band structures and regulating charge transfer properties [138]. For example, when polarized BaTiO_3_ interfaces with TiO_2_ semiconductor, the polarized electrical field boosts the band bending level of the TiO_2_ at the interface (Fig. 6A) [139]. Consequently, photogenerated electrons and holes quickly separate in opposite directions due to the enhanced built-in electric field, leading to faster response times and enhanced photocurrent (Fig. 6B). Additionally, the dark current decreases significantly from 10^−10^ A to 10^−12^ A.

Pyro-effect refers to the phenomenon where light irradiation alters the temperature distribution within a material, consequently changing the charge distribution and generating voltage signals. Essentially, light-induced pyro-effect modifies charge distribution, yielding voltage signals. For instance, the wurtzite-structured ZnO, with its noncentrally symmetric structure, exhibits pyroelectric polarizations due to temperature variations across the 2D semiconductor. The mechanism of pyro-phototronic effect based on pn junction is illustrated in Fig. 6C and D [140]. When UV light illuminates ZnO nanorods, inducing a self-induced transient temperature rise, negative pyro-polarizations emerge at the heterojunction interface (Fig. 6C). According to Anderson’s model, both the CB and valence band (VB) of ZnO at the interface increase, boosting charge carrier transport across the heterojunction and resulting in a transient high output current (Fig. 6E). The temperature fluctuation diminishes rapidly, with the photocurrent gradually stabilizing [140]. Upon UV light cessation, ZnO nanorods experience a transient temperature decrease, leading to positive pyro-polarizations at the interface. This causes the CB and VB of ZnO to decrease, creating a “carrier trap” effect due to positive pyro-polarizations (Fig. 6D), thereby reducing the dark current.

The piezo-photoelectric effect refers to the phenomenon observed in certain crystals where the application of pressure induces charge separation, generating a potential difference. This potential difference can drive electron movement within the crystal, resulting in a photoelectric effect on the crystal surface [9,141–145]. Typically, these crystals lack a central axis of symmetry (Fig. 6F) , which is crucial for inducing the piezo-photoelectric effect. An et al. [142] demonstrated and investigated the piezo-phototronic effect in ZnO nanosheets for the first time. When compressive stress is applied to the nanosheet, the photocurrent increases proportionally with the applied pressure, with higher pressure resulting in higher photocurrent, as depicted in the inset of Fig. 6G. Upon compressive stress on the source and drain electrodes, the negative piezoelectric polarization charges induced by stress reduce the Schottky barriers of both contacts. This reduction facilitates the separation of electron-hole pairs, leading to a strong photoresponse.

### Electrode adjustment and selection

**Fig. 7.**
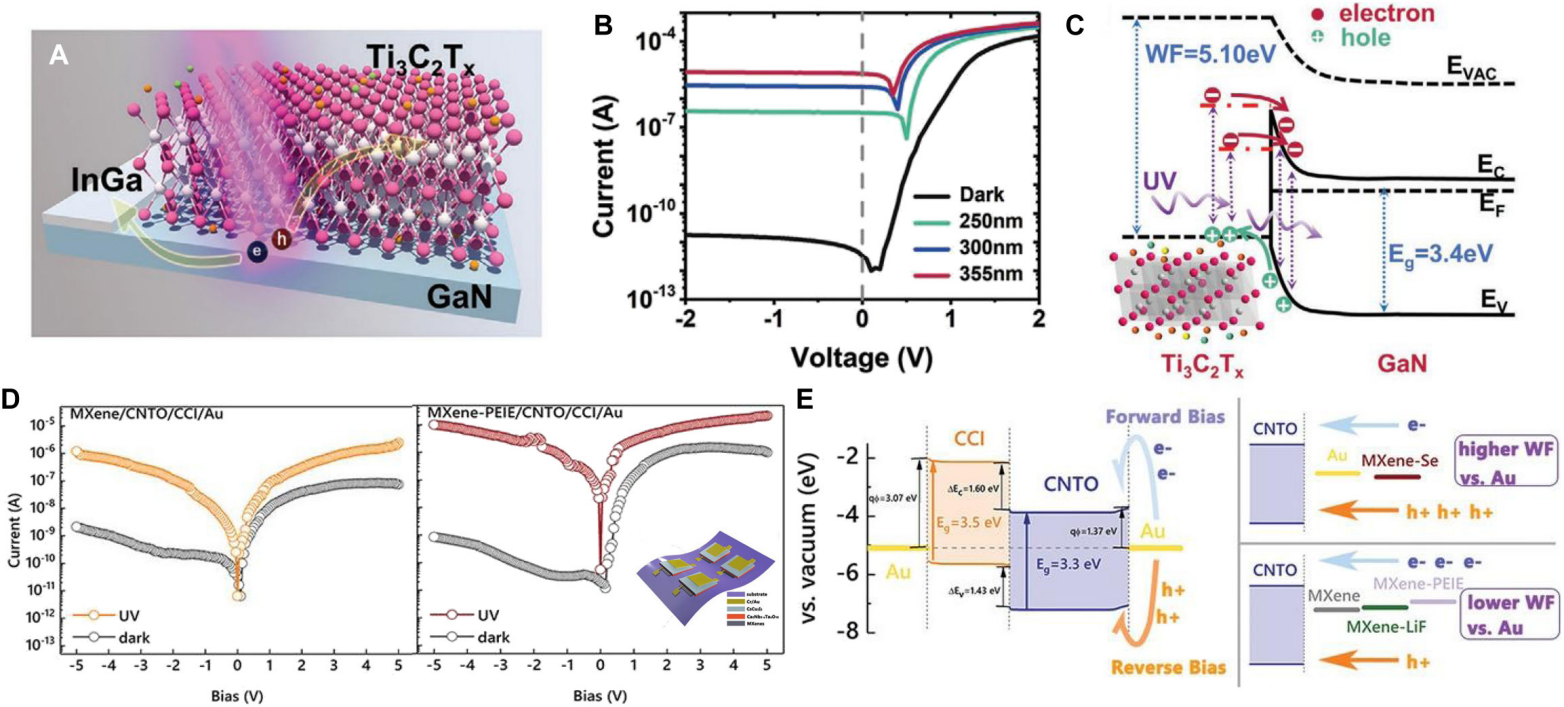
(A) Schematic of InGa-GaN-Ti_3_C_2_T_x_ UV PD. (B) *I*–*V* curves of the PD under dark and UV light. (C) Energy band diagram of Ti_3_C_2_T_x_/GaN heterojunction [76]. Copyright 2021 WILEY-VCH. (D) *I*–*V* curves of MXene/CNTO/CCI/Au and MXene-PEIE/CNTO/CCI/Au, and the inset is the corresponding schematic diagram of the PD. (E) Energy band diagram of Au/p-CsCu_2_I_3_/n-Ca_2_Nb_3−*x*_Ta*_x_*O_10_/MXene device [147]. Copyright 2022 WILEY-VCH.

Electrode work function refers to the minimum energy needed for electrons to escape from the semiconductor material upon contact with a metal electrode. It plays a vital role in the performance of semiconductor PDs. When incident photons irradiate the semiconductor, photogenerated carriers originate and need to be collected by the electrode to generate an electrical signal. However, if there is a significant difference between the electrode’s work function and the semiconductor’s CB or VB, the efficiency of charge carrier collection is greatly compromised, leading to PD performance degradation. Specifically, when the electrode’s work function exceeds that of the semiconductor’s CB, electron transfer from the semiconductor to the electrode becomes challenging, resulting in inefficient electron collection. On the contrary, if the work function of the electrode is lower than the VB maximum of the semiconductor material, it will be difficult for holes to transfer from the semiconductor to the electrode, resulting in low hole collection efficiency. Therefore, when designing semiconductor detectors, it is necessary to select appropriate electrode materials and processes to create an appropriate match between the electrode work function and the CB or VB of semiconductor materials, so as to improve the collection efficiency of charge carriers and improve the performance of detectors. Traditional metal and alloy electrodes, such as gold, silver, aluminum, indium gallium, titanium gold, and other electrodes, have already met most of our needs. Newly emerging electrode materials such as graphene and MXene have advantages that traditional electrodes do not have, such as transparency, flexibility, and adjustable electrode work functions, which are highly favored by researchers [146–148]. These electrodes have shown huge application prospects in the field of light detection. For example, Song et al. [76] demonstrated a self-powered InGa/GaN/MXene UV detector by using asymmetric electrode (Fig. 7A). Rectification characteristics are observed from Fig. 7B (*I*–*V* curves of the PD under dark and UV light), originating from the Schottky barrier formed at the metallic MXene/GaN interface. Under the light of 355-nm UV light, the PD shows an ultrahigh photocurrent of ~10^−5^ A with a responsivity of 284 mA/W. The energy band diagram of the MXene/GaN is illustrated in Fig. 7C. A strong built-in electric field formed at the interface between GaN and MXene because of the WF difference. Most of the electrons can penetrate through the MXene to the CB of GaN side under UV light, leading to the highly efficient UV light response. Furthermore, the work function of MXene electrode can be adjusted to match with semiconductor materials with different Fermi levels. With the surface modification of LiF, Se, and polyethylenimine ethoxylated (PEIE), the work function of Ti_3_C_2_T_x_ is tunned from 4.55 to 5.25 eV [147]. Vertical p-CsCu_2_I_3_/n-Ca_2_Nb_3−*x*_Ta*_x_*O_10_ junction UV PDs were fabricated by using MXene and modified MXene electrode. The results show that the PD shows enhanced rectification ratio (16,136) at ±2-V bias with PEIE decoration. The enhanced photocurrent can be clearly seen (Fig. 7D). The low work function of MXene/PEIE reduced the Schottky barrier between electrode and semiconductors, contributing to the higher performance of the PD (Fig. 7E).

## The Application of UV PDs

After extensive research and development, UV PDs have made significant advancements in performance. Their numerous advantages, including high signal-to-noise ratio, sensitivity, precision, and the ability to mitigate visible light interference, make them highly promising for applications in imaging, UV light communication, UV warning systems, and more. In the subsequent section, we will provide a comprehensive review of the progress of UV detectors across various application domains. This aims to deepen our understanding of the diverse applications of UV PDs and set the stage for future advancements in the field.

### UV PD for imaging

**Fig. 8.**
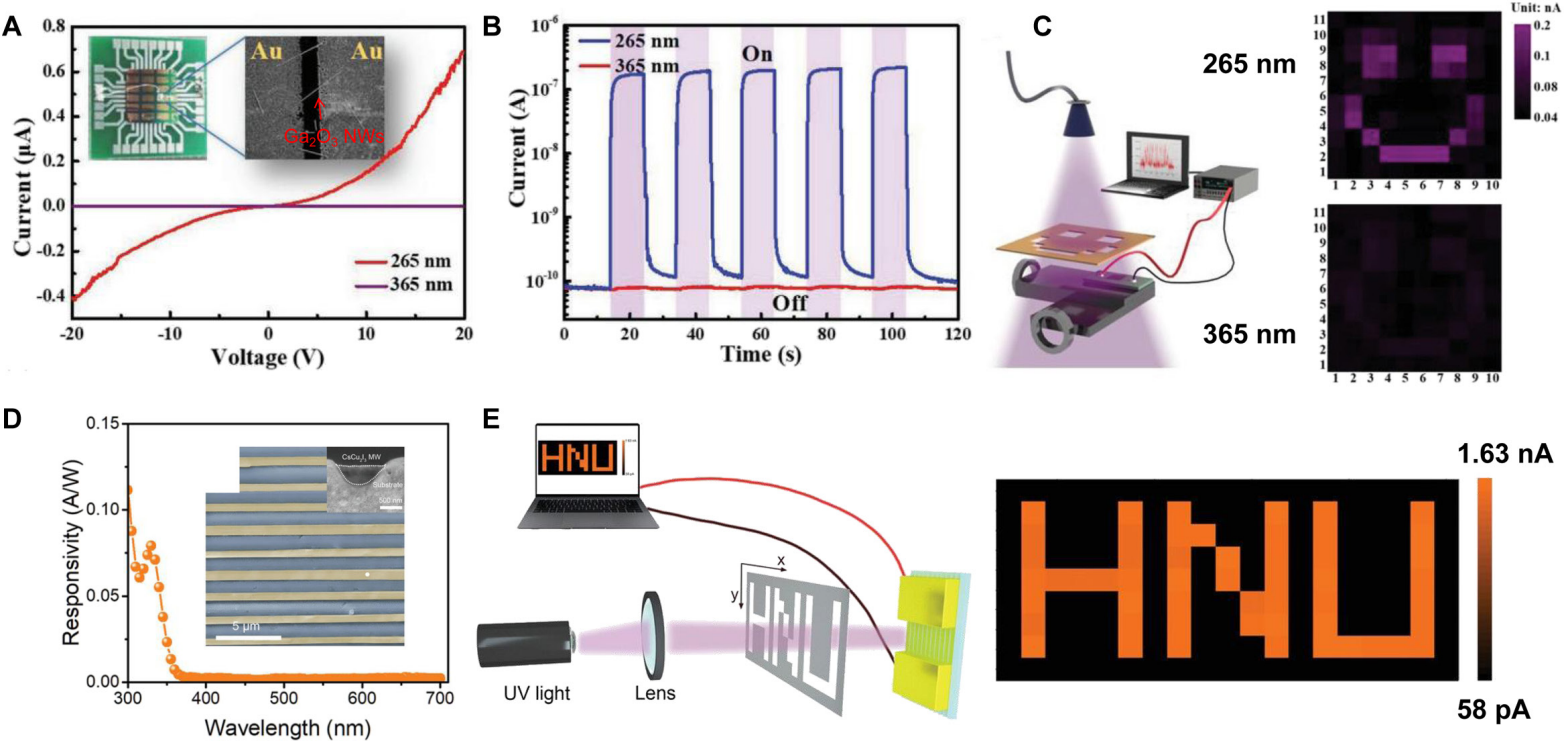
(A) *I*–*V* curves of the β-Ga_2_O_3_ nanowires (NWs) tested in dark and under 265- and 365-nm light. The insets are the optical and SEM image of the PD. (B) Corresponding *I*–*T* curves under dark and UV light at 10-V bias. (C) Schematic illustration of the imaging system (the PD moves in the *x* and *y* directions) and the corresponding 2D current mapping under 265- and 365-nm light, respectively [150]. Copyright 2019 WILEY-VCH. (D) Response spectrum of CsCu_2_I_3_-based PD, and the inset is the corresponding SEM images. (E) Schematic illustration of the imaging system (the mask moves in the *x* and *y* directions) and the corresponding 2D current mapping under 365-nm light [151]. Copyright 2022 WILEY-VCH.

The advantages of UV imaging mainly include the following. (a) It can penetrate certain transparent substances like smoke, fog, and clouds, allowing observation and imaging under their cover. (b) UV wavelengths enable the capture of high-resolution images due to their shorter wavelength compared to visible light. (c) Observation and imaging are feasible at night or under low light conditions, as strong UV radiation exists in the nocturnal sky and many substances exhibit significant spontaneous emission in the UV band. (d) UV imaging finds applications in studying various fields such as the atmosphere, geophysics, and biomedicine, where phenomena and substances exhibit unique characteristics and behaviors in the UV band [149]. Currently, common UV imaging methods include those based on single detectors and detector arrays. For example, the Luo group [150] developed a vapor–solid method without any catalyst for fabrication of β-Ga_2_O_3_ nanowires and constructed a Au/β-Ga_2_O_3_/Au UV PD (inset of Fig. 8A), which exhibited excellent deep UV detection ability, responding well to 265-nm light while barely responding to 365-nm light (Fig. 8A and B). To showcase its deep UV imaging prowess, they set up a UV imaging apparatus, depicted on the left side of Fig. 8C, where a mask featuring a smiling face was placed between UV light sources and the PD. By controlling the movement of the detector along the *x* and *y* axes, a series of different currents could be obtained. A clear 2D current mapping with 265-nm light as the source was discernible on the right side of Fig. 8C. Moreover, Xu et al. [151] reported a unidirectional CsCu_2_I_3_ microwire array-based UV PD with response spectrum below 350 nm (Fig. 8D). They demonstrated a single UV detector-based imaging system, depicted on the left side of Fig. 8E. Unlike the aforementioned UV imaging system, here the PD remained stationary, while the mask was manipulated along the *x* and *y* directions. UV light could pass through the mask’s hollowed-out positions, generating photocurrent detected by the PD, while the nonhollowed positions represented the dark current. These signals were visualized through a 2D current mapping, as illustrated on the right of Fig. 8E. Similar imaging equipment can also be used in solar-blind diamond-based MSM PDs [152].

**Fig. 9.**
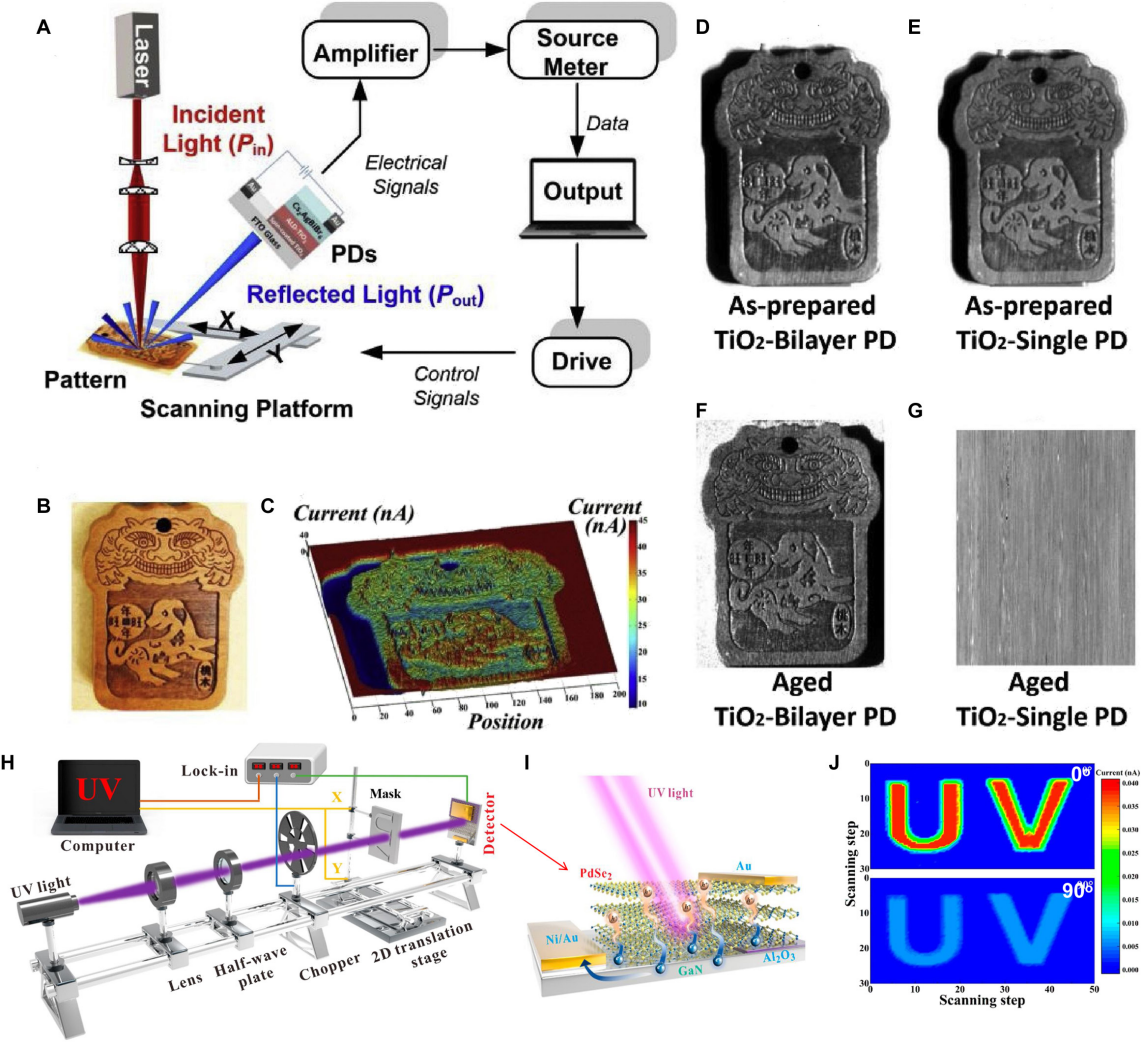
(A) Imaging system. (B) Pattern. (C) Light current distribution of using TiO_2_-bilayer PD. Images using (D) TiO_2_-bilayer PD and (E) TiO_2_-single PD. Images using (F) TiO_2_-Bilayer PD and (G) TiO_2_-single PD (aged 6 h) [153]. Copyright 2020 Elsevier Ltd. (H) Schematic of the imaging system of GaN/PdSe_2_ PD and the corresponding schematic diagram of the PD (I). (J) Imaging results under 360-nm light with polarization angles of 0° and 90° [154]. Copyright 2022 American Chemical Society.

In a pioneering study, the Mai group [153] explored the impact of a TiO_2_ bilayer film, prepared through a combination of atomic layer deposition and spin-coating processes on perovskite PDs [153]. This bilayer film not only enhanced UV stability but also reduced dark current. A focused laser scanning imaging system is shown in Fig. 9A, adopting a diffuse reflection imaging method. The diffuse reflected light is detected by the PD and then outputs the electrical signals (Fig. 9B and C) [153]. Comparative analysis revealed minimal difference between TiO_2_-bilayer and single-layer PDs (Fig. 9D and E). The TiO_2_-bilayer PDs exhibit high UV stability, maintaining high-quality imaging even after 6 h of UV exposure (Fig. 9F). Conversely, the TiO_2_-single PD failed to produce images after UV aging (Fig. 9G).

Additionally, polarized light, characterized by vibrations in specific directions, finds widespread applications in optics, electronics, communications, and biomedicine. Polarization-sensitive PDs hold promise for diverse fields, facilitating targeted object information acquisition. For example, the Jie group [154] demonstrated a self-powered PdSe_2_/GaN polarization-sensitive UV PDs, as shown in Fig. 9I. A single sensing pixel records the photocurrent as polarized UV light passes through a mask moving in the *X*-*Y* plane (Fig. 9H) [154]. Figure 9J exhibits the images of the “UV” letters (@ 360 nm) with polarization angles of 0° and 90°, respectively. The contrast ratio of 0° (1.4 × 10^3^) surpasses that of 90° (3.68 × 10^2^), revealing the PdSe_2_-based PD’s efficacy in polarized UV imaging [154].

**Fig. 10.**
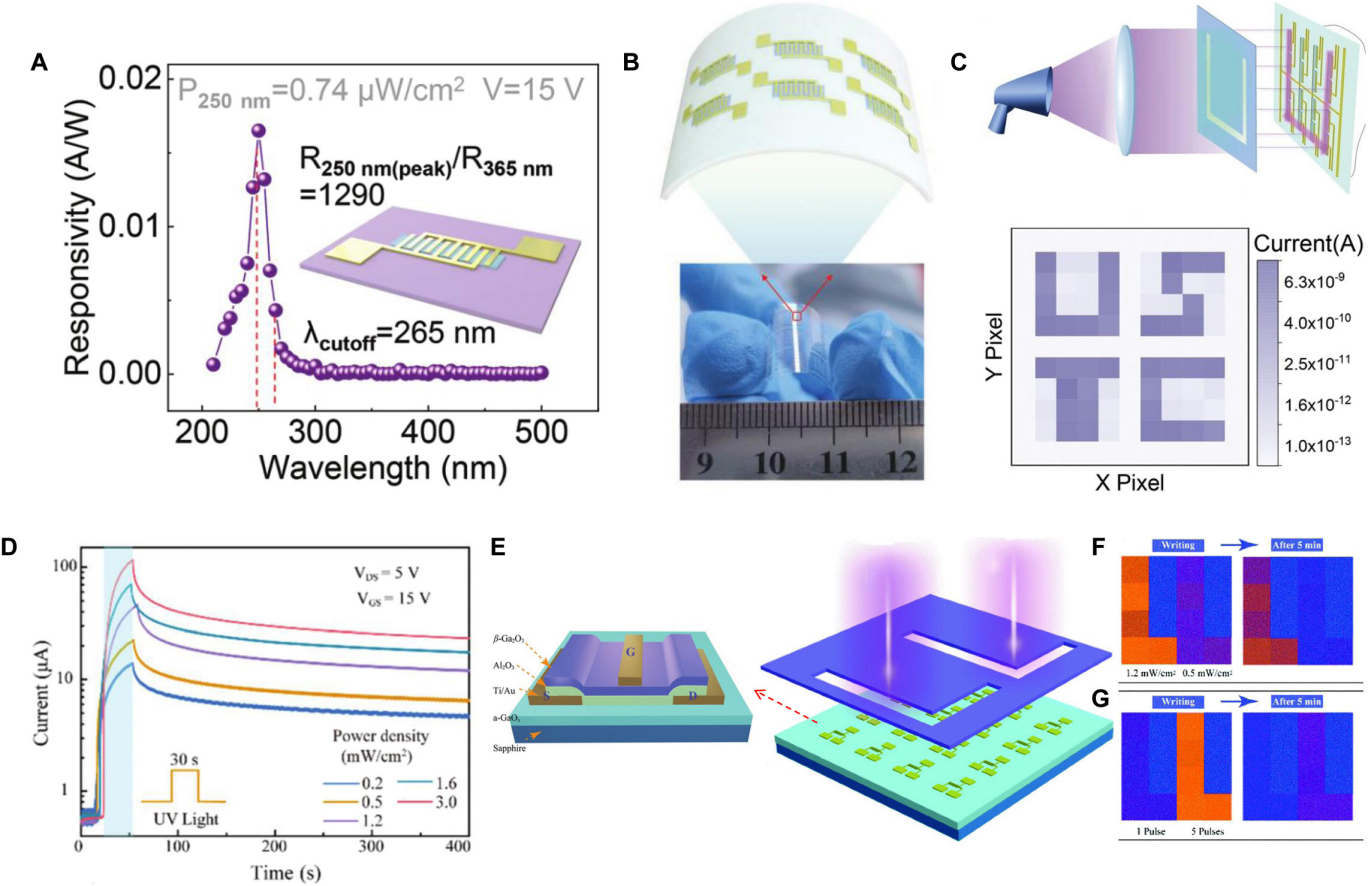
(A) Spectral responsivity of the device, and the inset is the corresponding schematic diagram. (B) Schematic and photograph of the device. (C) Diagrammatic sketch of the imaging system and the corresponding 2D mapping result [155]. Copyright 2022 WILEY-VCH. (D) Photocurrent as a function of the illumination intensity at VDS (drain and source voltage) = 5 V and VGS = 15 V (illumination period: 30 s). (E) Schematic illustration of the photomemory cell and the photomemory array. (F) Image storage results with intensity of 1.2 and 0.5 mW/cm^2^, respectively. (G) Image storage results with pulses of one and five times, respectively [157] .Copyright 2021 The Royal Society of Chemistry.

**Fig. 11.**
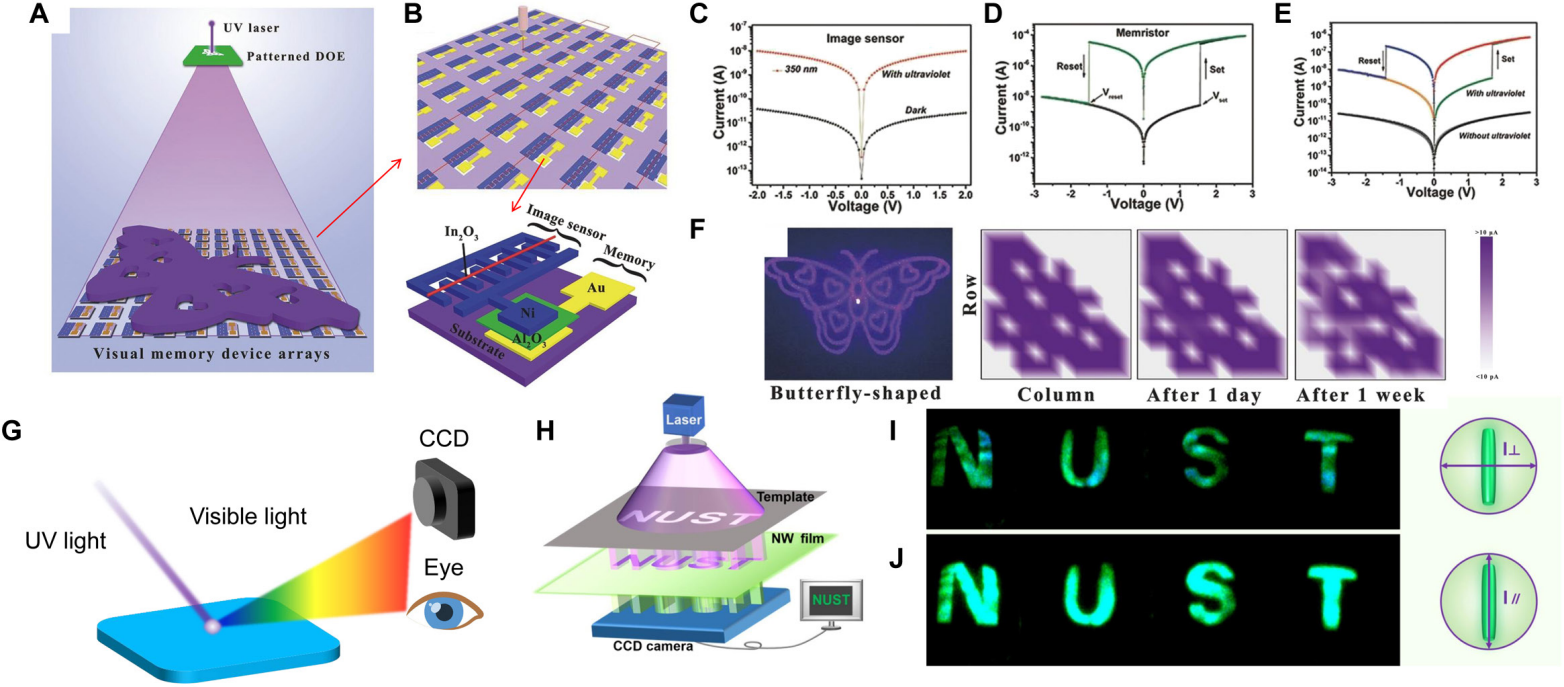
(A) Schematic illustration to detect and remember the butterfly UV information. (B) Corresponding enlarged view of the imaging and memory device. (C) Typical *I*–*V* curves of the image sensor under dark and 350-nm UV light. (D) *I*–*V* curves of the memristor. (E) *I*–*V* characteristic of a single device with and without UV light illumination. (F) Information storage behaviors of the flexible visual memory device arrays [159]. Copyright 2018 WILEY-VCH. (G) Schematic diagram of the UV light detect system. (H) UV imaging demonstration. (I and J) Letter pattern displayed when the polarization of the light source was perpendicular and parallel to the nanowires, respectively [160]. Copyright 2022 American Chemical Society.

The UV detector array enables simultaneous capture of multiple pixels, ideal for real-time imaging applications. Moreover, it seamlessly integrates with various electronic devices like computers, displays, and sensors. Its large-scale manufacturing process ensures uniform performance and characteristics across detectors, ensuring high repeatability. Long et al. [155] reported a flexible Ga_2_O_3_ DUV (deep ultraviolet) PDs based on optimized inkjet printing (Fig. 10B). At 15 V bias, the PDs demonstrate a peak responsivity at 250 nm and a cutoff responsivity at 265 nm with a *R*_250_/*R*_365_ rejection ratio of 1,290 (Fig. 10A). When the UV light passes through the template, a corresponding shape of UV beam will be generated (Fig. 10C) and the corresponding pixels will respond to the signals. So we can get the images immediately as shown at the bottom of Fig. 10C. Previous reports are almost focused on the sensing of UV light. To remember the imaging result, external circuits need to be added. Many approaches have been taken to realize the UV light memory ability by enhancing the decay time of UV PDs [156]. For example, the Shan group [157] combined the co-regulation of solar-blind light and VGS (grid voltage) with hole-trapping effects in the Ga_2_O_3_ phototransistor (left of Fig. 10E); a multilevel solar-blind photomemory array was demonstrated. The transistor realized a long time (>300 s) UV memory ability (Fig. 10D). The longer the UV irradiation time or the more irradiation times, the longer the UV imaging memory time (Fig. 10F and G). Even the Ga_2_O_3_ phototransistor realized UV image memory function for more than 5 min. However, compared to human long-term memory ability, this is still too short. Researchers have combined PDs with the nonvolatile resistive random-access memory (RRAM) devices to solve the abovementioned short time memory [158]. For instance, Chen et al. [159] integrated UV image sensor arrays with RRAM (Fig. 11A and B). The printed In_2_O_3_ nanowire UV PD owns a high on/off ratio of ~10^4^ (350 nm), and the RRAM device demonstrates a large memory window of >10^2^ (Fig. 11C to E). The stored information of the UV image can be maintained at least 1 week because of the nonvolatile characteristic of the RRAM device (Fig. 11F).

Recently, materials that can convert UV light to visible light are favored by researchers (Fig. 11G) due to its simple detection approach. For example, the Zeng group [160] demonstrated a strong polarized photoluminescence CsPbBr_3_ nanowire composite film. The CsPbBr_3_ nanowire composite film with polarization selective properties has the ability of converting the UVC light to green light, and the conventional charge-coupled device (CCD) camera or eyes can collect light imaging signals (Fig. 11H to J). The system gets over the deficiency of the CCD, making it hard for receiving the optical signal toward high-quality UV imaging.

### UV PD for communication

Apart from their imaging capabilities, UV detectors hold significant promise in optical communication. They offer advantages such as reduced loss, increased capacity, minimized interference, and adaptability to specialized environments. (a) Low loss: UV light’s shorter wavelength compared to visible light results in lower transmission losses through optical fibers, enabling longer signal transmission distances. (b) High capacity: The shorter wavelength of UV light allows for the transmission of more data within the same bandwidth, enhancing communication capacity. (c) Low interference: UV light’s short wavelength diminishes interference with visible light, enhancing signal stability and reliability in communication systems. (d) Suitable for special environments: UV light can penetrate certain transparent substances like smoke and clouds, making it suitable for applications in specialized environments where other wavelengths may encounter obstacles.

**Fig. 12.**
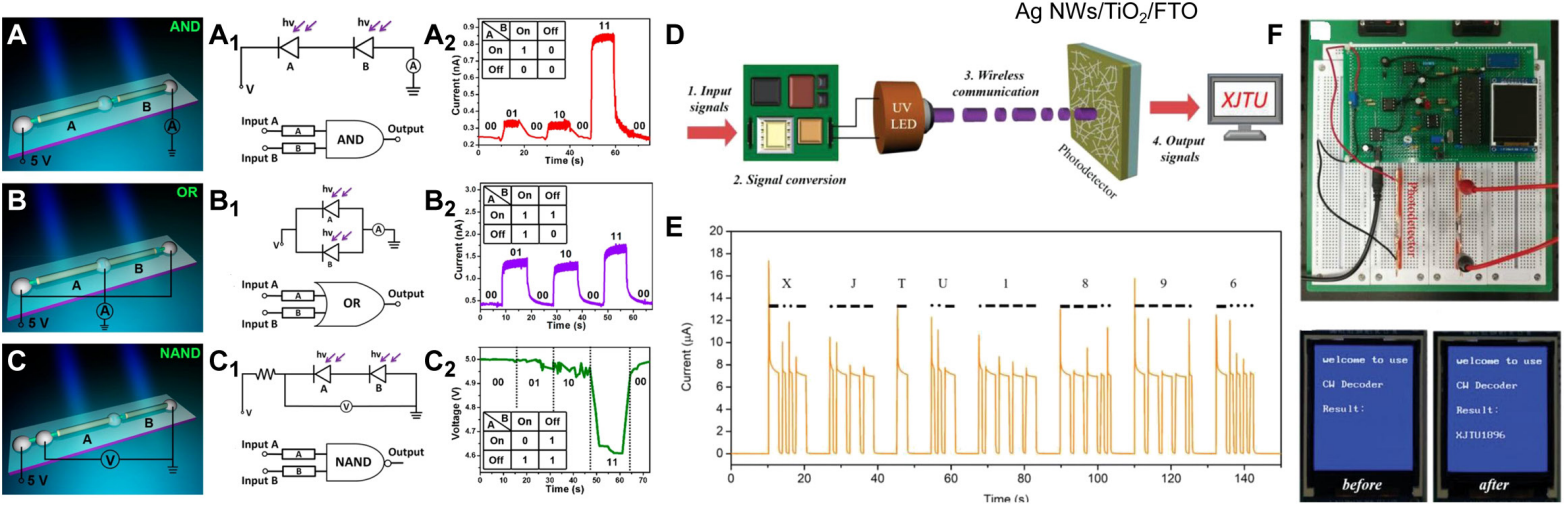
(A to C) Schematic diagram of different logic gates composed of two UV PDs. (A_1_, B_1_, and C_1_) Their corresponding schematic circuit diagrams. (A_2_, B_2,_ and C_2_) *I*–*T* curves for the circuit system under different illumination conditions (@ 5 V) [161]. Copyright 2019 American Chemical Society. (D) Schematic diagram of the UV communication system based on FTO/TiO_2_/Ag nanowire UV PD. (E) Morse code for “XJTU1896.” (F) Communication system and information of the getting signals [162]. Copyright 2019 WILEY-VCH.

In general, UV communication offers speed, reliability, and safety, encompassing point-to-point, multipoint, and reflective modes. Among these, point-to-point communication is extensively researched. For example, Zhang et al. [161] reported a flexible AZO/ZnO/PVK/PEDOT:PSS heterojunction on hair. Photon-triggered logic circuit can be achieved by the combination of the PDs, including AND (Fig. 12A and A1), OR (Fig. 12B and B1), and NAND gates (Fig. 12C and C1). By adjusting the signal of incident UV light, we can get different electrical signals, representing “00,” “01,” “10,” and “11,” respectively (Fig. 12A2, B2, and C2). Fang et al. [162] stacked a TiO_2_ film with FTO (SnO_2_:F) and Ag nanowire electrodes, forming asymmetric Schottky junctions in the device. The PDs demonstrate a responsivity of 32.5 mAW^−1^ (@ 0 V) and ultrafast response speed of 44 ns. Using self-powered FTO/TiO_2_/Ag as a signal receiver (Fig. 12D), a series of changing electrical signals can be obtained at the device terminal by orderly adjusting the UV pulse time (Fig. 12E). The information “XJTU1896” can be obtained when decoding the electrical signal by using Morse code (Fig. 12F).

Bispectral communication employs two distinct wavelength optical signals. Unlike traditional single-wavelength communication, bispectral communication offers several advantages. (a) Strong anti-interference ability: Utilizing two different wavelengths enables the system to switch wavelengths when encountering interference, thus enhancing communication stability and reliability. (b) Enhanced security: By utilizing different wavelengths, bispectral communication can implement encrypted communication, bolstering the security of data transmission. This feature is particularly valuable in scenarios where safeguarding sensitive information is paramount.

**Fig. 13.**
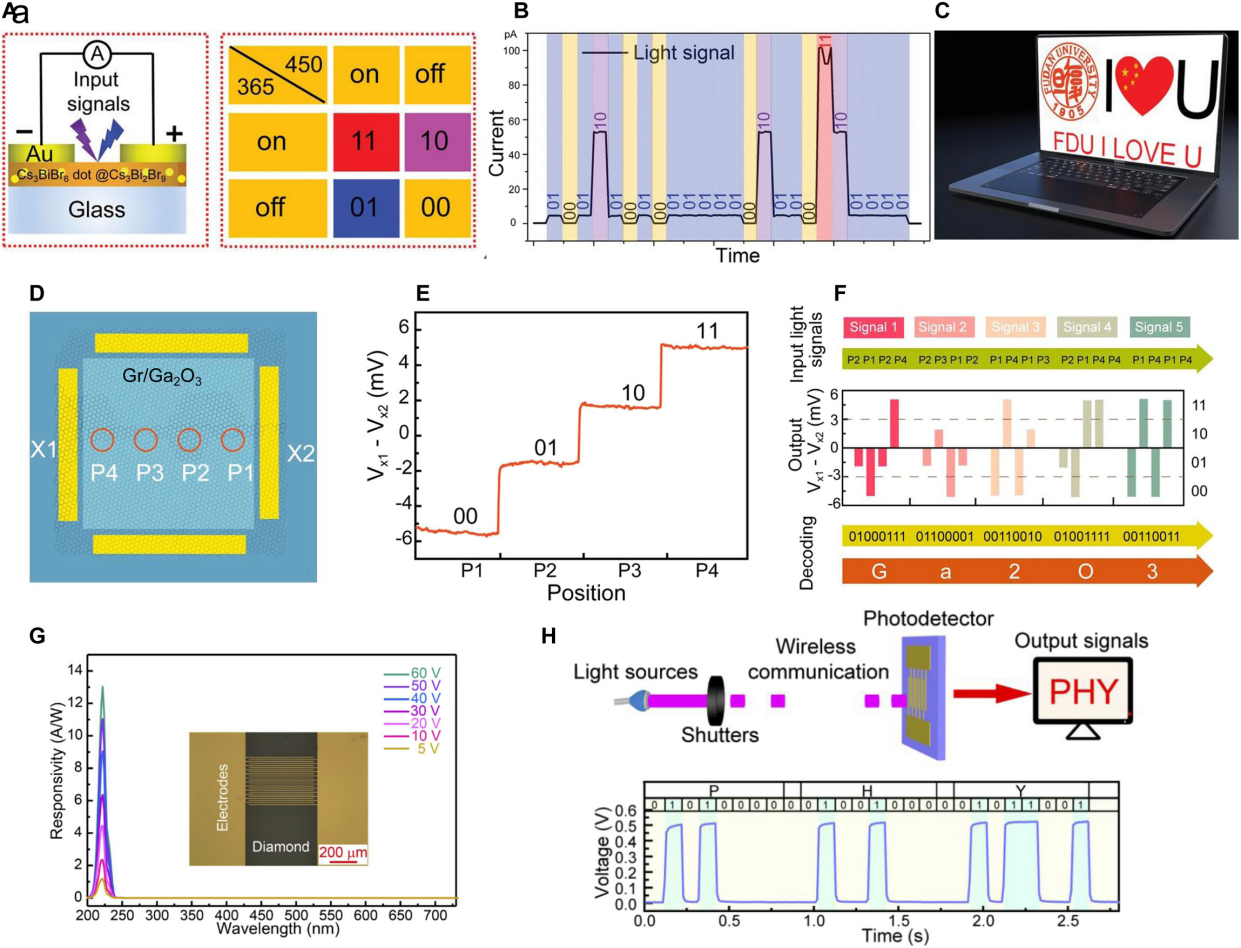
(A) Schematic diagram of Cs_3_Bi_2_Br_9_/Cs_3_BiBr_6_ BHJ PD and the corresponding signal generated under different light illumination. (B) Recorded light signals. (C) Transmitted signal [163]. Copyright 2022 WILEY-VCH. (D) Four points (P_1_, P_2_, P_3_, and P_4_) on the graphene/Ga_2_O_3_ (position-sensitive detectors) selected as the positions of the incident light. (E) Encoding the photo signals of P_1_, P_2_, P_3_, and P_4_ as “00,” “01,” “10,” and “11,” respectively. (F) Demodulating of the word “Ga_2_O_3_” according to the ASCII codes [164]. Copyright 2022 American Chemical Society. (G) Response spectra of the diamond MSM PD, and the inset is the optical images of the PD. (H) Light communication equipment and the corresponding recorded light signals [166]. Copyright 2019 Optical Society of America.

Recently, the Fang group [98,163] unveiled a Cs_3_BiBr_6_/Cs_3_Bi_2_Br_9_ bulk heterojunction (BHJ)-based dual-band PD (left of Fig. 13A). By adjusting the wavelength of the incident light (right of Fig. 13A), a series of changing electrical signals (Fig. 13B), which can be decrypted into “FDUIOU,” are obtained (Fig. 13C). Additionally, the Shan group [164,165] presented intriguing solar-blind position-sensitive detectors based on graphene/Ga_2_O_3_ Schottky junctions (Fig. 13D). When UVC light illuminates at P1, P2, P3, and P4, −5-, −2-, 2-, and 5-mV voltage differences (V_*x*1_ − V_*x*2_) are outputted, respectively, which can be recorded as “00,” “01,” “10,” and “11” signal, respectively ( Fig. 13E). By controlling the irradiation of UV light on different positions of the device, the signal “Ga2O3” shown in Fig. 13F was obtained. Their group [166] also demonstrated a diamond-based MSM UVC PD with a peak responsivity of 13 A/W (@ 222 nm; 60 V) ( Fig. 13G). It showed a great application prospects in wireless communication (Fig. 13H).

### UV PD for alarming

**Fig. 14.**
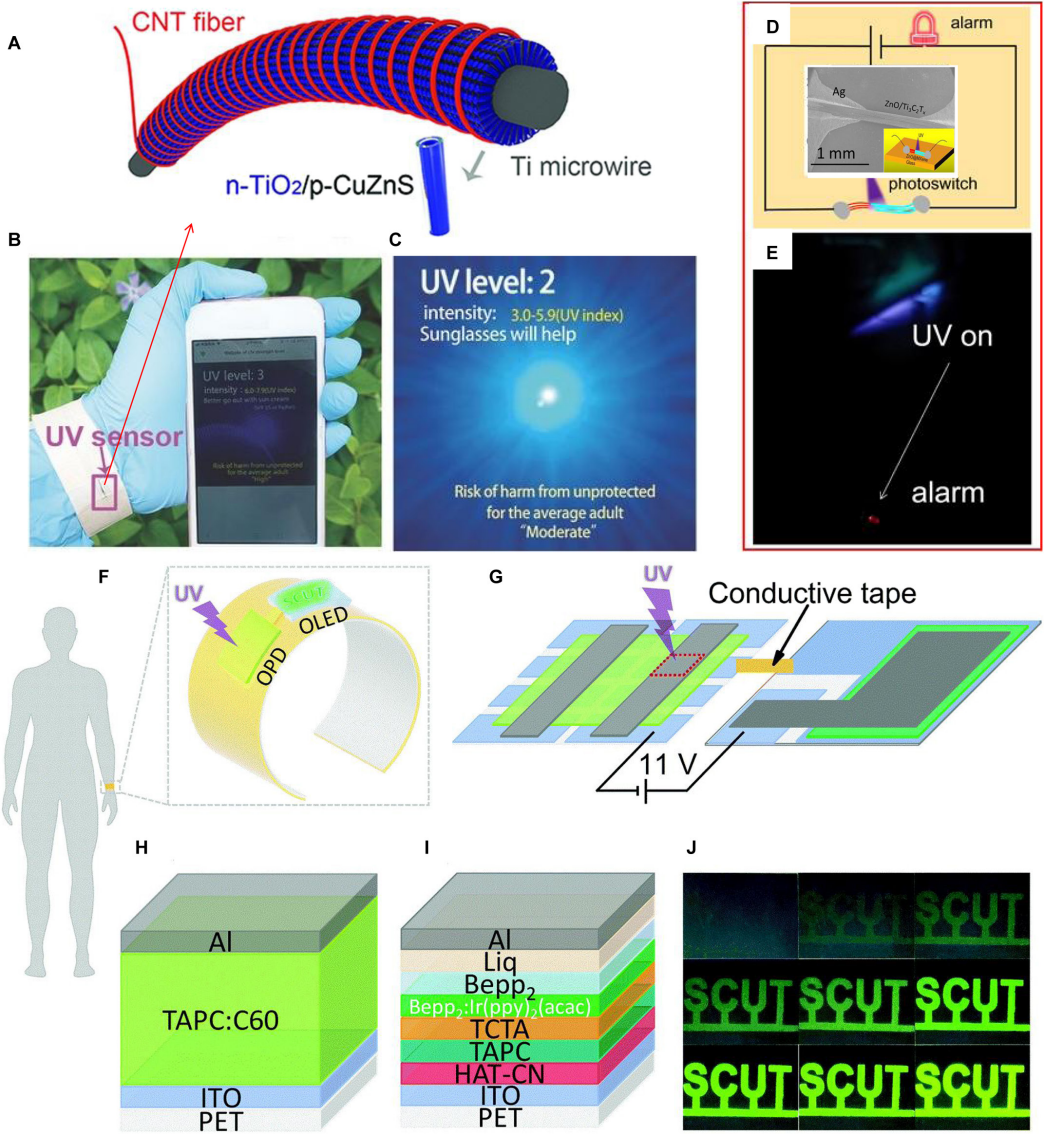
(A) Schematic illustration of the device CNT fiber/p-CuZnS/n-TiO_2_/Ti wire. (B and C) Photographs of a wearable UV sensor [167]. Copyright 2018 WILEY-VCH. (D) Circuit diagram of an alarming system, and the inset is an SEM image of ZnO/Ti_3_C_2_T_x_ micro-PD. (E) Photograph of the LED when the UV PD was illuminated [168]. Copyright 2021 AIP Publishing. (F) Schematic diagram of the wearable UV monitor. (G)` Composition of the UV monitor. Device structure of (H) flexible UV OPDs and (I) flexible OLEDs. (J) Alarming results of the wearable UV monitor under different UV light intensity [119]. Copyright 2021 The Royal Society of Chemistry.

In addition to their prevalent use in UV imaging and communication, UV PDs also hold significant importance in early warning and monitoring applications. Excessive UV radiation poses risks to human health and the environment, making UV PDs instrumental in monitoring and alerting about UV radiation intensity and exposure time to ensure human health and environmental safety. For example, UV PDs can monitor the damage of UV radiation to human skin, triggering alarms for protective measures. Similarly, they can monitor UV radiation’s impact on plants and provide early warnings about plant growth conditions. Various types of UV PDs are utilized to gauge UV light intensity and radiation levels. For instance, the Fang group [167] fabricated a flexible Ti/TiO_2_/CuZnS/CNT (carbon nanotube) fiber on Ti microwire as shown in Fig. 14A. The UV PD show a responsivity of 2.54 mA/W (@ 0 V, 300 nm). The UV PD shows wearable performance (Fig. 14B) and can indicate the intensity of UV radiation in real time (Fig. 14C). Our previous work [168] demonstrated a Ag/ZnO/MXene microwire UV PD on glass fiber (inset of Fig. 14D) by two steps dipping method. At 5-V bias and 365-nm UV light, the PD show a high responsivity (7.01 A/W) and outstanding EQE (2,386%). We designed a series circuit as shown in Fig. 14D. When applying 3-V bias, the red light-emitting diode (LED) cannot be brightened on dark condition. Once the 365-nm UV light irradiated on the PD, the increased photocurrent can bright the LED (Fig. 14E), achieving the purpose of UV alarming. Guo et al. [119] demonstrated an all-organic wearable (Fig. 14F and G) UV alarming system, which composed of an OPD (Fig. 14H) and a flexible organic LED (OLED) (Fig. 14I). With UV light illuminated on the OPD, the OLED is brightened and the brightness of OLED increases with the intensity of UV light (Fig. 14J).

### UV PD for other applications

**Fig. 15.**
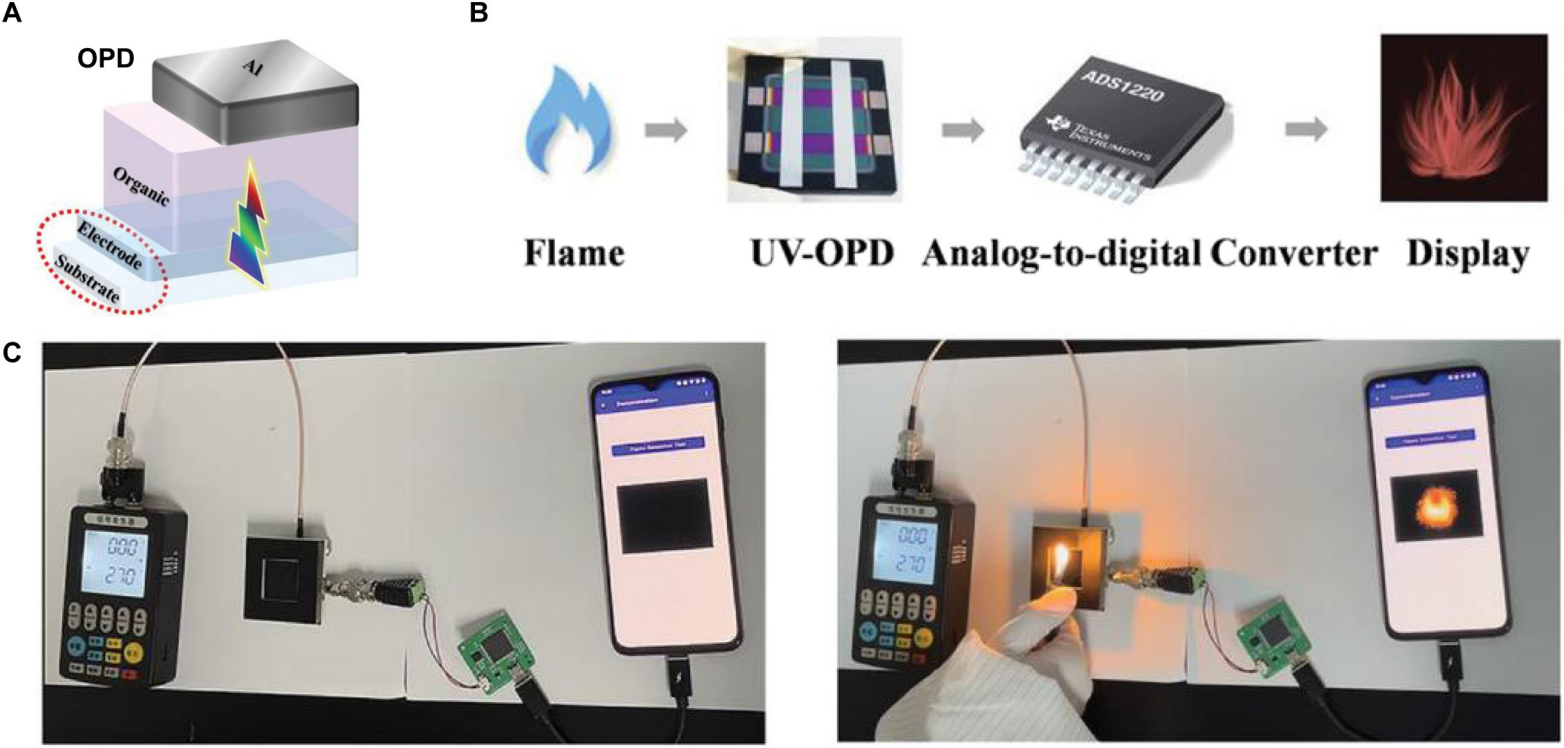
(A) Schematic diagram of the visible-blind UV PD. (B and C) Schematic illustration and camera graph of the flame detection system [118]. Copyright 2022 WILEY-VCH.

Apart from their established roles in UV imaging, communication, and early warning systems, UV PDs find extensive utility in high-voltage leakage detection and fire early warning applications [118,169]. UV PDs serve as effective flame detectors due to the emission of strong UV light during combustion. These short UV wavelengths are readily detectable by UV PDs. Flame detectors are typically installed in fire-prone areas such as kitchens and chemical laboratories. Upon detecting a flame, the PD promptly triggers an alarm to alert individuals to take preventive measures against fire hazards. The Ma group [118] reported a visible-blind UVB and UVC photomultiplication organic PD (OPD) by using wide bandgap tris(4-carbazoyl-9-ylphenyl)amine (TCTA) donor and fullerene-C_60_ acceptor BHJ, and the OPD is shown in Fig. 15A. The OPD shows high EQE as high as 195,665% at 255 nm. The photocurrent generated by the OPD when illuminated by fire was first converted to digital signal by an analog-to-digital converter (Fig. 15B), and then the phones received the signal. Finally, the fire can be warned in the mobile phone (Fig. 15C).

## Summary and Perspectives

**Fig. 16.**
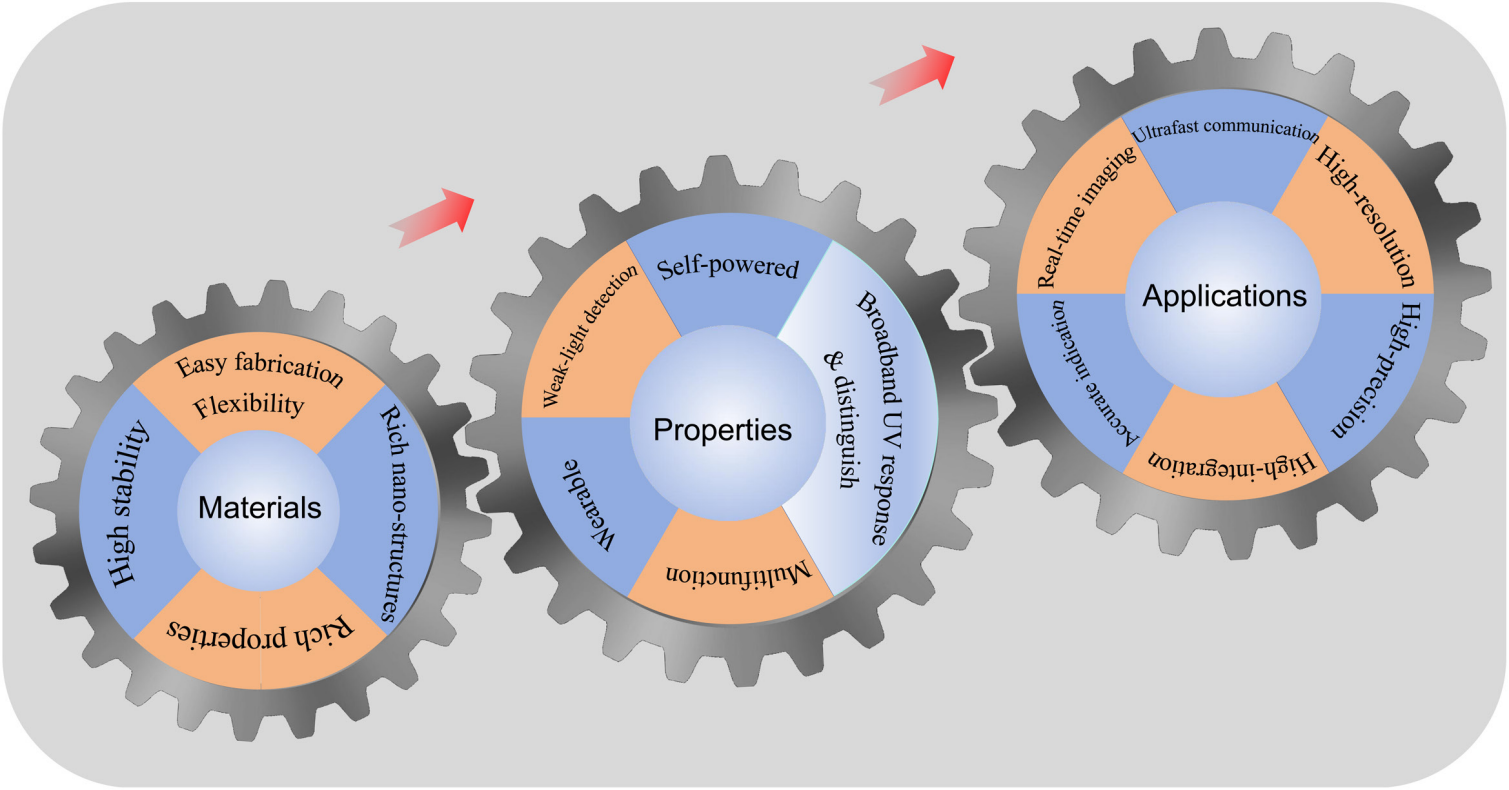
The development trend of the wide bandgap semiconductor-based UV PDs.

The development of wide bandgap semiconductors affords them being up-and-coming for UV PD application. This review provides a comprehensive overview of ongoing research on wide bandgap UV PDs and their diverse applications, encompassing both conventional wide bandgap semiconductors (ZnO, GaN, TiO_2_, Ga_2_O_3_, etc.) and emerging materials like perovskites and certain organic compounds. Despite considerable strides in wide bandgap semiconductor UV PD research, several challenges persist. (a) Commercialization of GaN, SiC, and diamond UV PDs has been achieved, but fabrication costs remain prohibitively high. The established methods for commercial GaN production, like molecular beam epitaxy and vapor phase epitaxy, necessitate costly equipment. Additionally, GaN’s poor compatibility with silicon substrates complicates its integration into existing integrated circuits. Similarly, the fabrication of SiC and diamond requires high temperatures and pressures, further adding to production costs. (b) Metal oxide (e.g., ZnO, TiO_2_, SnO_2_, and SNO)-based UV PDs face challenges, particularly regarding response speed, attributed to oxygen defects. Previous studies have highlighted the issue of surface absorption of O^2−^ or oxygen vacancies within the crystal structure, impacting device performance. Furthermore, exposure to UV light can induce changes in device behavior through the absorption and desorption of O^2−^ ions. (c) The poor stability of perovskite (CsCu_2_I_3_, Cs_3_Cu_2_I_5_, CsAg_2_I_3_, etc.) and organic materials-based UV PDs hinders their broader application. Perovskite and organic materials are prone to oxidation or degradation upon exposure to oxygen and water vapor in the atmosphere. Additionally, these materials exhibit susceptibility to aging under UV irradiation. Moving forward, the next phase of UV PD development should focus on addressing these challenges. Figure 16 illustrates potential directions for further advancement.

Considering materials for UV PDs, several key factors are crucial. (a) High stability: Stability is paramount for effective UV PD performance. This encompasses both thermodynamic stability, ensuring that materials remain stable at operating temperatures, and chemical stability, preventing reactions with surrounding environments such as oxygen and steam. Strong thermodynamic stability is essential, especially considering the heat generated during continuous UV light exposure. (b) Easy fabrication: Simplified fabrication methods offer numerous advantages, including cost reduction, easy process control, broad application potential, and scalability. Streamlined fabrication processes enable mass production of devices, fostering wider adoption and accessibility. (c) Rich nanostructure: The material’s structure profoundly influences its properties. A rich nanostructure enhances material performance, application versatility, stability, and other advantages. Nanomaterials with diverse morphologies exhibit varying physical and chemical properties, catering to different application requirements across sectors like catalysts, sensors, and optoelectronic devices. Moreover, different nanostructures entail distinct surface energies and active sites, influencing material stability and durability, critical for advancing high-performance and stable UV detectors. (d) Flexibility: Flexible materials possess high flexibility and plasticity, encompassing bending, tensile, and torsional properties. They offer adaptability to surfaces with varying shapes and curvature, ensuring good adhesion. Additionally, their lightweight and soft nature enables superior comfort and conformity to the human body’s movements. Flexibility represents a significant direction in material science and industrial manufacturing for UV PDs, promising enhanced usability and performance. (e) Rich properties: Different applications require different material properties to fulfill specific requirements. For example, the aerospace field needs the semiconductor materials not only with good electrical and photoelectric properties but also with high temperature resistance and corrosion resistance.

From the perspective of properties: (a) Weak light detection: In practical applications, UV signal reaching detectors often weaken over long distances, resulting in diminished electrical signals. Enhancing photon utilization involves designing nanostructures to capture more incident UV light and integrating plasmonic noble metal NPs with PDs to generate additional hot electrons during UV exposure. Simultaneously, minimizing noise interference through circuit processing is crucial. (b) Self-powered operation: The emphasis on energy conservation and environmental preservation drives the adoption of self-powered UV detectors. Operating without external bias voltage, they offer benefits such as independence from power supplies, streamlined system architecture, energy efficiency, wide applicability, and low maintenance costs. Constructing high-quality p-n, p-i-n, or n-n junctions enables self-powered UV PDs. (c) Wearable design: Wearable UV PDs, characterized by their compactness, lightweight nature, comfort, portability, and user-friendliness, facilitate real-time monitoring of UV radiation intensity to prompt appropriate protective measures. Utilizing wide bandgap 2D semiconductors or wide bandgap quantum dot semiconductors meets the requirements for flexible and wearable UV PDs. (d) Broadband UV response and UV wavelength differentiation. It is crucial for a single PD to discern between UVA, UVB, and UVC rays due to their distinct functionalities. Achieving this requires the PD to respond across the entire UV spectrum. However, most PDs are limited to specific UV regions due to semiconductor bandgap effects. Combining various wide bandgap semiconductors offers a viable solution for creating broadband UV PDs. (e) Multifunction: As integrated circuits evolve, there is a growing demand for multifunctional devices. These devices not only shrink in size, saving space and enhancing efficiency, but also streamline design and boost reliability. UV PDs with sensing, memory, and computational capabilities are gaining attention. Combining UV PDs with RRAM devices enables such functionality. Additionally, polarized UV detection holds promising applications. Integrating wide bandgap semiconductors with polarization-absorbing materials like CuS or PdSe_2_ opens avenues for polarized UV detection.

From the perspective of applications: (a) Real-time imaging: Current single-device UV imaging technology is slow, scanning pixel by pixel, unsuitable for real-time tracking needs such as rocket tracking and high-voltage leakage detection. Future UV PDs should aim for real-time imaging to monitor and track objects in real time by monitoring their UV radiation. (b) Ultrafast communication: Although UV communication has a very broad application prospect, the following problems still need to be overcome in order to realize its ultrafast communication: First, more advanced UV light source and UV optical fiber are needed to achieve higher transmission speed and greater bandwidth. Second, the transmission loss and attenuation of UV signal need to be improved in UV communication. Lastly, as the receiver of the signal, the speed of the UV PD still needs to be improved. (c) Accurate indication: Accurate detection of UV light is crucial for environmental monitoring, industrial processes, medical diagnoses, and safety precautions. For instance, (a) Environmental monitoring: Accurate indication of UV light can help monitor the intensity and distribution of UV light in the environment so as to improve the accuracy and reliability of environmental monitoring. (b) Industrial production: The accurate indication of UV can help the UV processing and detection in industrial production, such as UV curing and UV etching. (c) Medical diagnosis: Accurate instructions of UV rays can help doctors diagnose and treat skin diseases such as vitiligo. (d) Safety protection: The accurate indication of UV rays can help people understand the harm of UV rays so as to take corresponding safety protection measures, such as wearing anti-UV glasses and applying sunscreen. (e) Advancing integration, resolution, and precision: UV PDs must align with the development trajectory of integrated circuits. Thus, achieving high levels of integration, resolution, and precision is imperative for their evolution.
